# Screening of Bioactive Microalgae from Freshwaters, Collected in Hue, Vietnam: Cytotoxic Constituents from *Dolichospermum smithii* HU04

**DOI:** 10.3390/molecules31010165

**Published:** 2026-01-01

**Authors:** Nguyen Thi Minh Hang, Nguyen Thi Thu Ha, Hoang Duc Manh, Duong Thi Thuy, Hoang Thi Quynh, Nguyen Thi Thu Lien, Nguyen Thi Tu Oanh, Tran Huu Giap, Buu Huu Tai, Doan Thi Mai Huong, Ngo Quoc Anh, Nguyen Xuan Nhiem

**Affiliations:** 1 Institute of Chemistry, Vietnam Academy of Science and Technology, 18 Hoang Quoc Viet, Nghia Do, Hanoi 10072, Vietnam; minhhang@ich.vast.vn (N.T.M.H.); thuha.vast@gmail.com (N.T.T.H.); nguyentuoanh11101976@gmail.com (N.T.T.O.); huugiap@ich.vast.vn (T.H.G.); bhtaiich@gmail.com (B.H.T.); huongdm@ich.vast.vn (D.T.M.H.); ngoqanh@ich.vast.vn (N.Q.A.); 2 National Foundation for Science and Technology Development, Ministry of Science and Technology, 113 Tran Duy Hung, Yen Hoa, Hanoi 10072, Vietnam; hdmanh@mst.gov.vn; 3 Institute of Science and Technology for Energy and Environment, Vietnam Academy of Science and Technology, 18 Hoang Quoc Viet, Nghia Do, Hanoi 10072, Vietnam; duongthuy0712@gmail.com (D.T.T.); quynhphuonghae@gmail.com (H.T.Q.); 4 Institute of Applied Research for Science and Technology, University of Sciences, Hue 530000, Vietnam; nthuliencnsh@gmail.com; 5 Graduate University of Science and Technology, Vietnam Academy of Science and Technology, 18 Hoang Quoc Viet, Nghia Do, Hanoi 10072, Vietnam

**Keywords:** *Dolichospermum smithii*, cyanobacteria, smithioside A, smithioside B, phenolic glycoside, cytotoxic activity

## Abstract

Background/Objectives: Microalgae are recognized as prolific producers of bioactive metabolites with pharmaceutical potential. This study aimed to isolate and characterize cytotoxic constituents from selected cytotoxic microalgae, collected in Hue city, Vietnam. Methods: Microalgal samples were collected from freshwater bodies, morphologically identified, and maintained in laboratory culture. Thirteen strains were successfully isolated and cultivated in BG11, Z8, and BBM media to determine optimal growth conditions. Cytotoxic effects of extracts/compounds were determined using the sulforhodamine B assay on human lung cancer (SK-LU-1) and human liver cancer (HepG2) cell lines. The methanol extract was partitioned with *n*-hexane and CH_2_Cl_2_, followed by extensive chromatographic separation and HPLC purification to afford twelve compounds, including two new and ten known compounds. The structures were elucidated by HR-ESI-MS and NMR spectra, chemical methods, and comparing compounds in the literature. Results: From the phytoplankton samples collected across six freshwater bodies in Hue city, Vietnam, thirteen microalgal strains were successfully isolated and purified under laboratory conditions. These strains were morphologically and taxonomically identified to be *Microcystis aeruginosa* HU05, *Microcystis viridis* HU13, *Anabaena circinalis* HU08, *Aphanizomenon flos-aquae* HU02, *Dolichospermum smithii* HU04, *Calothrix braunii* HU14, *Nostoc muscorum* HU12, *Nostoc punctiforme* HU11, *Raphidiopsis raciborskii* HU03, *Lyngbya spiralis* HU15, *Planktothrix stagnina* HU16, *Phormidium subtilis* HU06, and *Scenedesmus quadricauda* HU07. All methanol extracts of those microalgae were evaluated for cytotoxic activity. The MeOH extracts of *M. viridis* (HU13) and *D. smithii* (HU04) exhibited significant cytotoxic effects, with IC_50_ values of 6.19 ± 0.80 and 4.89 ± 0.76 µg/mL for *M. viridis*, and 9.51 ± 0.84 and 8.32 ± 0.94 µg/mL for *D. smithii* against SK-LU-1 and HepG2 cell lines, respectively. Furthermore, chemical studies of *D. smithii* HU04 led to the isolation of two new compounds, smithioside A (**1**) and smithioside B (**2**) and ten known ones, 3,4,5-trimethoxyphenyl-1-*O*-β-D-glucopyranoside (**3**), 4′-hydroxy-3′-methoxyphenol-β-D-[6-*O*-(4″-hydroxy-3″,5″-dimethoxylbenzoate)]-glucopyranoside (**4**), 4′-hydroxy-2′,6′-dimethoxyphenol 1-*O*-β-D-(6-*O*-syringoyl)glucopyranoside (**5**), mallophenol B (**6**), pisoninol II (**7**), guaiacylglycerol (**8**), (*E*)-asarone (**9**), deacetylsarmentamide B (**10**), (*E*)-2-hexenyl-β-D-glucopyranoside (**11**), and 5,6-dihydropyridin-2(1*H*)-one (**12**). The cytotoxic activity of all isolated compounds was also evaluated against SK-LU-1 and HepG2 cancer cell lines. Compound **12** showed the strongest activity, with IC_50_ values of 9.13 ± 0.89 µM (SK-LU-1) and 7.64 ± 0.46 µM (HepG2). Compounds **5** and **6** exhibited moderate cytotoxic activity on both human cancer cell lines with IC_50_ values ranging from 25.99 to 51.47 µM. Conclusions: These results highlight the potential of *Dolichospermum smithii* HU04 as a source of bioactive compounds, particularly in anticancer applications. These findings suggest that *D. smithii* HU04 extracts could be developed for therapeutic purposes targeting cancer.

## 1. Introduction

Freshwater microalgae are ubiquitous across rivers, lakes, reservoirs, wetlands, and benthic biofilms, where they function as foundational primary producers that regulate nutrient cycling, support aquatic food webs, and deliver wide-ranging ecosystem services with societal relevance [1]. Although decades of bioprospecting have emphasized marine systems, the chemical space of freshwater microalgae remains comparatively less surveyed in many regions, representing a timely opportunity to uncover novel scaffolds and mechanisms. Methodologically, contemporary discovery pipelines couple taxonomic authentication (integrative morphology with DNA barcodes and environmental sequencing) to axenic culture with metabolite-inducing stress regimens, and then apply green extraction; subsequently, orthogonal fractionation [2]. Microalgae have been known as one of the largest and most diverse photosynthetic organisms. They are potential sources for bioactive compounds such as fatty acids, carotenoids, chlorophylls, phycobiliproteins, and vitamins. The compounds from microalgae have exhibited antitumor, anti-inflammatory, antibacterial, antifungal, and antiviral activities [3]. This paper reports on the isolation, identification and cytotoxic screening of extracts and isolated compounds from selected microalgae.

## 2. Results and Discussion

### 2.1. Sampling Sites and Environmental Characteristics

**Figure 1:**
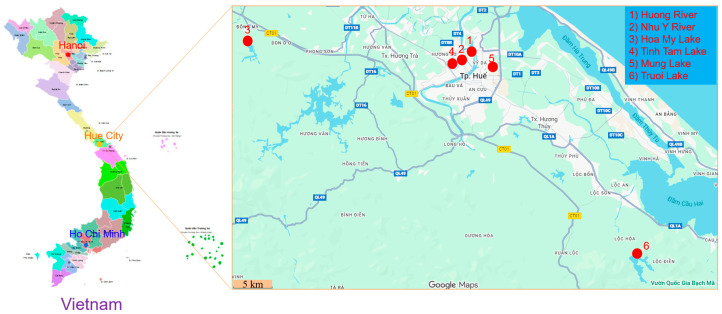
The study area (Vietnam map and sampling stations in Hue city).

Microalgal samples were collected from six representative freshwater bodies in Hue city, Central Vietnam (Figure 1). These locations were selected to cover a diversity of hydrological and environmental conditions, including urban rivers and natural or man-made lakes that receive varying degrees of anthropogenic influence. The sampling sites comprised: (1) Huong River, (2) Nhu Y River, (3) Hoa My Lake, (4) Tinh Tam Lake, (5) Mung Lake, and (6) Truoi Lake.

Huong river is the main river flowing through Hue city, characterized by a moderate current and seasonal fluctuation in nutrient levels. The river receives moderate inputs from domestic and agricultural effluents and frequently experiences cyanobacterial blooms during the dry season. Sampling stations (SH1–SH5): SH1 (16.3980, 107.5750); SH2 (16.4510, 107.5460); SH3 (16.4640, 107.5820); SH4 (16.3138, 107.3428); SH5 (16.5480, 107.6145).

Nhu Y river is a small tributary of the Huong River, flowing through densely populated residential areas. This river segment is characterized by slow water movement and high levels of organic matter, creating eutrophic conditions favorable for filamentous cyanobacteria such as *Planktothrix* and *Dolichospermum*. Sampling sites (NY1-NY5): NY1 (16.4639, 107.6151), NY2 (16.4726, 107.6157), NY3 (16.4739, 107.6075), NY4 (16.4847, 107.6390), and NY5 (16.4845, 107.6225).

Hoa My lake is a shallow artificial lake situated in the northern urban area of Hue city. It receives surface runoff and aquaculture effluents from nearby communities. The lake’s eutrophic nature, with pH ranging from 6.8 to 7.6, provides favorable conditions for dense growth of planktonic algae and cyanobacteria. Sampling sites (HM1-HM5): HM1 (16.4990, 107.3170), HM2 (16.4982, 107.3203), HM3 (16.4956, 107.3181), HM4 (16.4925, 107.3234), and HM5 (16.4896, 107.3235).

Truoi lake is a large freshwater reservoir located approximately 30 km south of Hue city. It serves as a key water resource for irrigation and domestic use. The reservoir represents a relatively unpolluted ecosystem with clear water, stable pH (6.5–7.2), and high dissolved oxygen content (5.2–6.5 mg/L). Sampling sites (HT1-HT5): HT1 (16.2556, 107.7847), HT2 (16.2557, 107.7873), HT3 (16.2496, 107.7882), HT4 (16.2389, 107.7911), and HT5 (16.2476, 107.7987).

Mung lake is a small semi-urban freshwater body surrounded by agricultural land. Mung lake receives runoff rich in nutrients and organic matter, resulting in moderate eutrophication that promotes the growth of *Microcystis*, *Nostoc*, and *Anabaena*. Sampling sites (MN1-MN5): MN1 (16.4793, 107.5806), MN2 (16.4792, 107.5811), MN3 (16.4786, 107.5811), MN4 (16.4788, 107.5806), and MN5 (16.4789, 107.5809).

Tinh Tam lake is an ornamental lake located within the Imperial Citadel of Hue. It has limited water circulation and is enriched with organic debris from surrounding vegetation. Sampling sites (TT1-TT5): TT1 (16.4780, 107.5756), TT2 (16.4785, 107.5773), TT3 (16.4770, 107.5763), TT4 (16.4766, 107.5749), and TT5 (16.4774, 107.5759).

**Figure 2:**
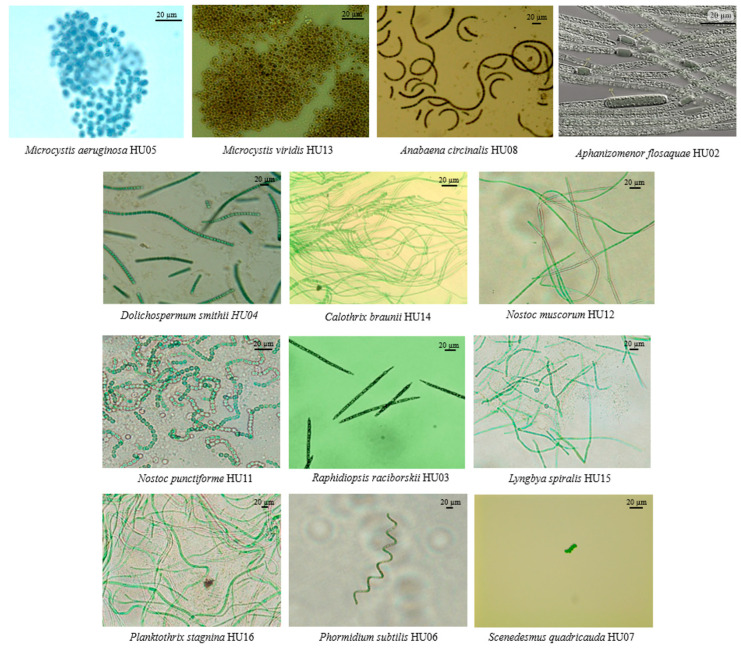
Morphological Characteristics of Microalgal strains Isolated from Freshwater Bodies in Hue City, Vietnam.

From the phytoplankton samples collected across six freshwater bodies in Hue city, thirteen microalgal strains were successfully isolated and purified under laboratory conditions. These strains were morphologically and taxonomically identified into eleven genera belonging to two algal divisions, cyanobacteria (12 species) and chlorophyta (1 species) (Table 1) [4]. Morphological characteristics of microalgal strains have been shown in Figure 2.

### 2.2. Optimization of Culture Media for Small-Scale Cultivation of Microalgae

Small-scale culture experiments (5 L) were conducted to determine the optimal growth media for thirteen microalgal species isolated from freshwater bodies in Hue city. Each strain was cultivated in 5 L Erlenmeyer flasks containing 3 L of culture medium under controlled laboratory conditions (25 ± 2 °C, light intensity 1500–2000 lux, 12:12 h light–dark cycle, and gentle aeration with filtered air). Three standard freshwater media, BG11, Z8, and BBM, were tested for their ability to support biomass accumulation and stable growth.

The results showed that BG11 and Z8 media supported the best growth performance for most cyanobacterial strains, while BBM was more suitable for the green alga *Scenedesmus quadricauda* HU07. Among the heterocystous cyanobacteria, *Dolichospermum smithii* HU04, *Anabaena circinalis* HU08, and *Aphanizomenon flos-aquae* HU02 grew optimally in Z8 medium, reaching exponential phase after 8–10 days. *Microcystis aeruginosa* HU05 and *Microcystis viridis* HU13 exhibited robust planktonic growth in BG11 medium, while filamentous non-heterocystous species such as *Raphidiopsis raciborskii* HU03, *Lyngbya spiralis* HU15, *Phormidium stagnina* HU16, and *Planktothrix subtilis* HU06 also developed dense trichomes in the same medium. Benthic strains *Nostoc punctiforme* HU11 and *Nostoc muscorum* HU12 adhered strongly to the vessel walls and formed characteristic mucilaginous colonies in BG11. In contrast, the green alga *Scenedesmus quadricauda* HU07 displayed limited growth in cyanobacterial media but produced the highest cell density in BBM medium, consistent with its Chlorophyta physiology. Overall, BG11 was confirmed as the most suitable culture medium for small-scale biomass production of cyanobacteria, whereas Z8 provided a favorable alternative for heterocystous genera such as *Dolichospermum* and *Anabaena* (Table 2).

### 2.3. Large-Scale Cultivation of D. smithii HU04

Under the optimized conditions, Z8 medium; nitrogen supplied as NaNO_3_ at 150%; phosphorus supplied as KH_2_PO_4_ at 200% of the Z8 standard; 10% inoculum (starter ~10^6^ cells/mL); pH 7.5; continuous aeration; 22–25 °C; 3000–4000 lux—*D. smithii* HU04 (flasks (150 L) reached harvest at day 10. Across the production run, a total of 2.0 Kg dry biomass was obtained, corresponding to ~19.5 g per flask (≈1.30 g/L), providing ample material for extraction, isolation, and bioassays (Table 2).

### 2.4. Cytotoxic Evaluation of Microalgal Extracts

The cytotoxic activities of methanol extracts obtained from thirteen microalgal species isolated in Hue city were evaluated against two human cancer cell lines, SK-LU-1 and HepG2 (Table 2). Among the tested samples, the MeOH extracts of *M. viridis* (HU13) and *D. smithii* (HU04) exhibited significant cytotoxic effects, with IC_50_ values of 6.19 ± 0.80 and 4.89 ± 0.76 µg/mL (*M. viridis*), and 9.51 ± 0.84 and 8.32 ± 0.94 µg/mL (*D. smithii*) against SK-LU-1 and HepG2 cell lines, respectively. The remaining extracts displayed weak or no activity (IC_50_ > 100 µg/mL). These results suggest that certain cyanobacteria from Hue freshwater ecosystems produce cytotoxic metabolites with promising anticancer potential. Similar findings have been reported in other cyanobacterial genera. *Microcystis* spp. are known to produce hepatotoxic and cytotoxic peptides such as microcystins and aeruginosins, which exhibit IC_50_ values ranging from 5–30 µg/mL against HepG2 and HeLa cells [5]. *Dolichospermum* species have also been recognized for producing bioactive compounds, including peptides and alkaloids with significant apoptosis-inducing effects [2]. The cytotoxic activity of *D. smithii* HU04 is consistent with earlier reports that cyanobacteria belonging to the Nostocales order possess diverse secondary metabolites (phenolic glycosides, cyclic peptides, and alkaloid derivatives) with antitumor and antimicrobial properties [2]. These results highlight the importance of freshwater cyanobacteria from Vietnam as a potential source of pharmacologically active compounds. Overall, *M. viridis* HU13 and *D. smithii* HU04 were identified as the most promising candidates for further chemical investigation. The latter was selected for compound isolation and structural elucidation due to its higher biomass yield, balanced extraction efficiency (10.77%), and reproducible cytotoxic activity.

### 2.5. Structural Elucidation

**Figure 3:**
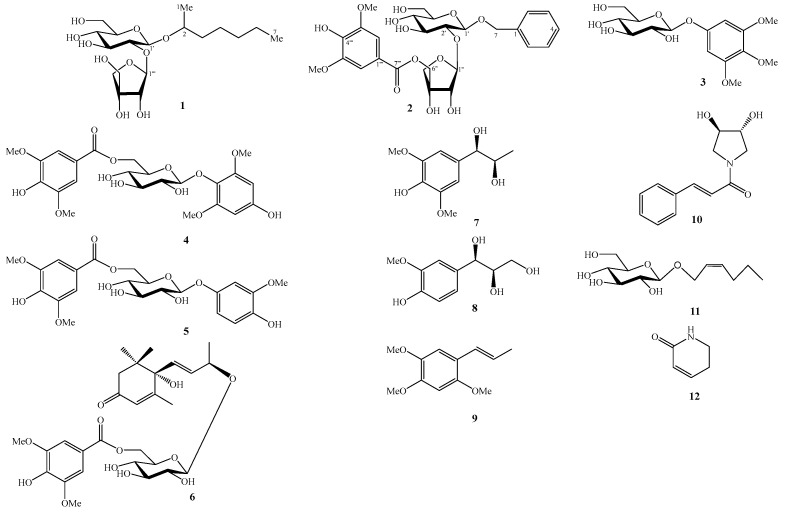
Chemical Structures of Compounds **1**–**12**.

The known compounds were determined to be 3,4,5-trimethoxyphenyl-1-*O*-β-D-glucopyranoside (**3**) [6], 4′-hydroxy-3′-methoxyphenol-β-D-[6-*O*-(4″-hydroxy-3″,5″-dimethoxylbenzoate)]-glucopyranoside (**4**) [7], 4′-hydroxy-2′,6′-dimethoxyphenol 1-*O*-β-D-(6-*O*-syringoyl)glucopyranoside (**5**) [8], mallophenol B (**6**) [9], pisoninol II (**7**) [10], guaiacylglycerol (**8**) [11], (*E*)-asarone (**9**) [12], deacetylsarmentamide B (**10**) [13], (*E*)-2-hexenyl-β-D-glucopyranoside (**11**) [14], and 5,6-dihydropyridin-2(1*H*)-one (**12**) [15] by comparing their NMR data with those reported in the literature (Figure 3).

**Figure 4:**
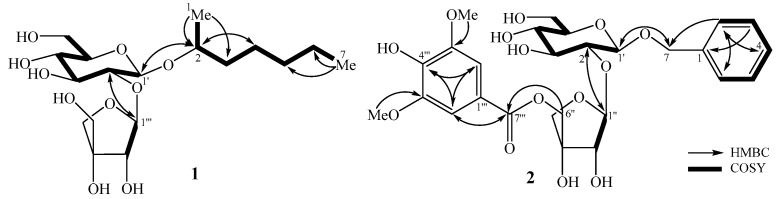
The key HMBC and COSY correlations of Compounds **1** and **2**.

Compound **1** was obtained as a white amorphous powder. Its molecular formula was determined to be C_18_H_34_O_10_ based on pseudo-ion peak at *m*/*z* 411.2220 [M+H]^+^ on the HR-ESI-MS (Calcd. for [C_18_H_35_O_10_]^+^, 411.2225) and the ^13^C-NMR spectrum. The ^1^H-NMR spectrum of **1** showed signals of two anomeric protons at *δ*_H_ 5.41 (1H, d, *J* = 1.8 Hz) and 4.41 (1H, d, *J* = 7.8 Hz), two methyl groups at *δ*_H_ 1.18 (3H, d, *J* = 6.0 Hz) and 0.93 (3H, t, *J* = 6.6 Hz). The ^13^C-NMR spectrum of **1** exhibited signals of 18 carbons, comprising one non-protonated carbon at *δ*_C_ 80.8, eight methines at *δ*_C_ 110.5, 100.7, 78.8, 78.6, 78.0, 77.7, 75.3, and 71.9, seven methylenes at *δ*_C_ 75.4, 66.3, 62.9, 38.4, 33.1, 26.1, and 23.7, and two methyl carbons at *δ*_C_ 19.6 and 14.4 (Table 3). Analysis of ^1^H- and ^13^C-NMR spectra indicated that **1** contains two monosaccharide units and a heptane-2-ol unit, similar to oroxylumoside A, except for the difference of aglycone relative to the literature analogue [16]. The HMBC correlations from H-1 (*δ*_H_ 1.18) to C-2 (*δ*_C_ 75.3)/C-3 (*δ*_C_ 38.4), from H-2 (*δ*_H_ 3.90) to C-1 (*δ*_C_ 19.6)/C-3 (*δ*_C_ 38.4)/C-4 (*δ*_C_ 26.1), and from H-7 (*δ*_H_ 0.93) to C-5 (*δ*_C_ 33.1)/C-6 (*δ*_C_ 23.7) supported the presence of heptan-2-ol. Acid hydrolysis of **1** furnished D-apiose and D-glucose as sugar components (identified as trimethylsilyl (TMS) derivatives by a gas chromatography (GC) method). The HMBC correlations between api H-1″ (*δ*_H_ 5.41) and glc C-2′ (*δ*_H_ 78.6), between glc H-2′ (*δ*_H_ 3.33) and api C-1″ (*δ*_H_ 110.5), and between glc H-1′ (*δ*_H_ 4.41) and C-2 (*δ*_H_ 75.3) were observed (Figure 4). These observations suggested the sequence of sugar linkages to be D-apiofuranosyl-(1→2)-β-D-glucopyranoside and sugar moiety located at C-2 of heptane-2-ol. Accordingly, the structure of **1** was determined as heptan-2-ol 2-*O*-β-D-apiofuranosyl-(1→2)-β-D-glucopyranoside, a new compound named smithioside A.

Compound **2** was obtained as a white amorphous powder and its molecular formula, C_27_H_34_O_14_, determined on the basis of HR-ESI-MS at *m*/*z* 581.1855 [M-H]^−^ (Calcd. for [C_27_H_33_O_14_]^−^, 581.1875). The ^1^H-NMR spectrum of **2** showed the signals of five protons of monosubstituted phenyl ring at *δ*_H_ 7.34 (2H, d, *J* = 7.8 Hz), 7.22 (2H, t, *J* = 7.8 Hz), and 7.17 (1H, t, *J* = 7.8 Hz), two aromatic protons at *δ*_H_ 7.36 (2H, s), and two anomeric protons at δ_H_ 4.40 (1H, d, *J* = 7.8 Hz) and 5.01 (1H, d, *J* = 2.4 Hz), indicating the presence of two sugar units. The ^13^C-NMR and HSQC spectra of **2** displayed 27 carbons, of which, 7 carbons were assigned to a benzyl unit at *δ*_C_ 71.9, 129.2 × 4, 128.6, and 138.9; 9 carbons to a syringyl unit at *δ*_C_ 57.0 × 2, 108.6 × 2, 121.0, 143.0, 149.1 × 2, and 168.0; and 11 carbons to two monosaccharide residues at *δ*_C_ 62.8, 66.8, 71.5, 75.4, 78.0, 78.5, 78.6, 78.9, 79.2, 102.2, and 110.4. Comparison of these data with hattushoside [17] indicated the absence of the *p*-hydroxy group on the phenyl ring relative to that reference. The ^13^C-NMR data of **2** are consistent with one β-apiofuranosyl and one β-glucopyranosyl unit [18]. The HMBC correlations between H-1″ (*δ*_H_ 5.44) and C-2′ (*δ*_C_ 78.9), and between H-2′ (*δ* 3.50) and C-1″ (*δ* 110.4) established the linkage β-D-apiofuranosyl-(1″→2′)-β-D-glucopyranoside. In addition, HMBC correlations between H-2/H-6 (*δ*_H_ 7.34) and C-4 (*δ*_C_ 128.6)/C-7 (*δ*_C_ 71.9), H-3/H-5 (*δ*_H_ 7.22) and C-1 (*δ*_C_ 138.9)/C-4 (*δ*_C_ 128.6) supported the presence of a benzyl unit; HMBC correlations between H-2‴/H-6‴ (*δ*_H_ 7.36) and C-3‴/C-5‴ (*δ*_C_ 149.1)/C-4 (*δ*_C_ 143.0)/C-7‴ (*δ*_C_ 168.0), and between the methoxy protons (*δ*_H_ 3.90) and C-3‴/C-5‴ (*δ*_C_ 149.1), confirmed presence of a syringyl unit (Figure 4). The HMBC correlations between H-7 (*δ*_H_ 4.55 and 4.91) and C-1 (*δ*_C_ 102.2) and between H-6″ (*δ*_H_ 4.27 and 4.40) and C-7‴ (*δ*_C_ 168.0) indicated the positions of benzyl and syringyl units at C-1′ and C-6″, respectively. Based on the above evidence, the structure of **2** was elucidated as benzyl-(5-*O*-syringyl)-β-D-apiofuranosyl-(1→2)-β-D-glucopyranoside and named smithioside B.

### 2.6. Cytotoxic Evaluation

The cytotoxicity of compounds **1**–**12** isolated from the biomass of *Dolichospermum smithii* HU04 was evaluated against two human cancer cell lines, SK-LU-1 and HepG2 (Table 4). As results, compound **12** showed the strongest activity, with IC_50_ values of 9.13 ± 0.89 µM (SK-LU-1) and 7.64 ± 0.46 µM (HepG2), similar to those of a positive control, ellipticine (IC_50_ values of 3.51 ± 0.38 and 3.42 ± 0.45 µM, respectively). Compounds **5** and **6** exhibited moderate cytotoxic activity on both human cancer cell lines with IC_50_ values ranging from 25.99 to 51.47 µM. Compounds **4**, **9**, and **10** exhibited weak cytotoxic effects (IC_50_ values ranging from 61.05 to 84.06 µM. The remaining compounds **1**–**3**, **7**, **8**, and **11** were inactive at the tested concentrations (IC_50_ > 100 µM). Overall, HepG2 tended to be more sensitive than SK-LU-1, with consistently lower IC_50_ values for the active compounds. Cyanobacteria (including the *Anabaena*/*Dolichospermum*/*Aphanizomenon*) are prolific producers of cytotoxic secondary metabolites; phylogenomic and review studies highlight abundant biosynthetic gene clusters and numerous antitumor leads from cyanobacteria overall [19]. 2-Pyridone frameworks showed significant cytotoxic activity in HepG2 and other tumor lines [20]. (*E*)-Asarone derivative typically shows moderate cytotoxic activity on HepG2 cell line [21] and carries genotoxic/carcinogenic concerns, so it is best kept as a reference rather than a lead.

## 3. Material and Methods

### 3.1. General

All NMR spectra were recorded on a Bruker 500 MHz and 600 MHz (Billerica, MA, USA). Data processing was performed with MestReNova ver. 9.0.1. HR-ESI-MS spectra were obtained using a Waters ACQUITY UPLC system (Milford, MA, USA) connected to a Xevo G2-XS QTOF at the Korea Basic Science Institute (KBSI, Metropolitan Seoul Center). Column chromatography (CC) was performed on silica-gel (Kieselgel 60, 230–400 mesh, Merck, Darmstadt, Germany) or RP-18 resins (30–50 μm, Fuji Silysia Chemical Ltd., Okazaki-shi, Japan). For thin layer chromatography (TLC), pre-coated silica-gel 60 F254 (0.25 mm, Merck) and RP-18 F254S (0.25 mm, Merck) plates were used.

### 3.2. Sampling and Sample Collection

Phytoplankton samples were collected from a range of freshwater environments across Hue province, central Vietnam. These included ponds, lakes, reservoir and rivers. Surface water samples were obtained using a 40 μm mesh plankton net and transferred into sterile 500 mL polyethylene bottles. Samples were stored at 4 °C, shaded containers and transported to the laboratory.

### 3.3. Isolation Strains

Isolation of microalgal strains was performed using a micropipette isolation technique under an inverted microscope (Olympus CK40, Tokyo, Japan). The isolation was carried out with BG11 (Blue-Green algae medium) [22,23], Z8 [24] and BBM (Bold’s Basal Medium) [25] culture media. The BG11 medium contained the following components (mg/L): NaNO_3_, (1.500); K_2_HPO_4_, (30.5); MgSO_4_.7H_2_O, (75); CaCl_2_·2H_2_O, (36); C_6_H_8_O_7_, (6); (NH_4_)_5_[Fe(C_6_H_4_O_7_)_2_], (6); Na_2_EDTA-Mg, (1); and Na_2_CO_3_, (20). In addition, 1 (mL/L) of an A5 trace-metal solution was added, consisting of (mg/L): H_3_BO_3_ (2.860); MnCl_2_·4H_2_O (1.810); ZnSO_4_·7H_2_O (222); Na_2_MoO_4_·2H_2_O (390); CuSO_4_·5H_2_O (79); and Co(NO_3_)_2_·6H_2_O (49). The Z8 medium having the following chemical ingredients (mg/L): MgSO_4_·7H_2_O (25); NaNO_3_ (467); Ca(NO_3_)_2_·4H_2_O (59); K_2_HPO_4_·3H_2_O (41); Na_2_CO_3_ (21); EDTA-Na_2_ (3.705); FeCl_3_ (2.8); Gaffron micronutrients, 1 mL (Gaffron micronutrients having the following chemical ingredients (mg/L): Na_2_WO_4_·2H_2_O (3.3); (NH_4_)_6_Mo_7_O_24_·2H_2_O (8.8); KBr (12); KI (8.3); ZnSO_4_·7H_2_O (28.7); Cd(NO_3_)_2_·4H_2_O (15.5); Co(NO_3_)_2_·6H_2_O (14.6); CuSO_4_·5H_2_O (12.5); NiSO_4_(NH_4_)_2_SO_4_·6H_2_O (19.8); Cr(NO_3_)_3_·9H_2_O (4.1); V_2_O_5_ (8.9); KAl(SO_4_)_2_·12H_2_O (47.4); H_3_BO_3_ (310); MnSO_4_·4H_2_O (22.3) plus water to 1 L). The BBM medium contained the following components (mg/L): NaNO_3_ (250); MgSO_4_·7H_2_O (75); NaCl (25); K_2_HPO_4_ (75); KH_2_PO_4_ (175); CaCl_2_·2H_2_O (25); H_3_BO_3_ (11.4); ZnSO_4_·2H_2_O (8.82); MnCl_2_·4H_2_O (1.44); MoO_3_ (0.71); CuSO_4_·5H_2_O (1.57); Co(NO_3_)_2_·6H_2_O (0.49); EDTANa (50); KOH (31); FeSO_4_·7H_2_O (4.98); and H_2_SO_4_ (0.001 mL). Under an inverted microscope (Olympus CK40), individual filaments or cells were isolated from phytoplankton samples using sterile glass Pasteur pipettes. The isolates were rinsed in several drops of sterilized culture medium to remove contaminating cells or suspended particles, and then transferred onto sterile agar plates containing BG11, Z8, or BBM (Bold’s Basal Medium). The plates were incubated under fluorescent light (1500–2000 lux) at 25 ± 3 °C with a 12:12 h light/dark photoperiod. Once a distinct single colony appeared on the agar plate, it was aseptically transferred onto a fresh agar medium for subculturing. This procedure was repeated four to five times to ensure the purity and stability of the isolated microalgal strains. The purified filaments or single colonies were then transferred into test tubes containing 10–20 mL of culture medium and incubated under the same conditions described above. The morphology of the isolated strains was examined using a light microscope (Olympus CX51) equipped with a charge-coupled device (CCD) camera for image capture. Identification of the isolated strains was based on their morphological features and performed according to the standard cyanobacterial and green algal taxonomic references, including [26,27]. The 13 strains included in this study were designated *Microcystis aeruginosa* HU05, *Microcystis viridis* HU13, *Anabaena circinalis* HU08, *Aphanizomenon flos-aquae* HU02, *Dolichospermum smithii* HU04, *Calothrix braunii* HU14, *Nostoc muscorum* HU12, *Nostoc punctiforme* HU11, *Raphidiopsis raciborskii* (HU03, *Lyngbya spiralis* HU15, *Planktothrix stagnina* HU16, *Phormidium subtilis* HU06, and *Scenedesmus quadricauda* HU07. Microalgae were preserved on agar plates at Institute of Science and Technology for Energy and Environment, Vietnam Academy of Science and Technology. In this method, cultures are grown on nutrient agar and stored at low temperature (4–10 °C) under low-light conditions to slow metabolic activity. During storage, periodic monitoring is required to prevent contamination and loss of strain characteristics.

### 3.4. Batch Cultivation and Harvesting Microalgae for Screening Cytotoxic Effects

For bioactivity screening, a total of 13 isolated algal strains were cultured under controlled laboratory conditions. The isolated strains were cultivated in 1000 mL Erlenmeyer flasks containing 500 mL of standard media, including Z8 medium for *M. aeruginosa* HU05, *M. viridis* HU13, *A. flos-aquae* HU02, *N. muscorum* HU12, *N. punctiforme* HU11, *R. raciborskii* HU03, *C. braunii* HU14, *P. subtilis* HU06, *L. spiralis* HU15, and *P. stagnina* HU16, *A. circinalis* HU08, *D. smithii* HU04; and BBM medium for *S. quadricauda* HU07. All cultures were aerated with filtered air (pore size 0.22 µm) and maintained at 25 ± 3 °C in a temperature-controlled room under cool white fluorescent lamps (1500–2000 lux) with a 12:12 h light–dark cycle for 14 days. The culture suspension of each strain was harvested by centrifugation at 10,000 rpm for 10 min, and the collected biomass was stored at −20 °C for further analysis. Biomass of all thirteen algae were ultrasonically extracted with methanol (MeOH) at 40 °C three times with MeOH: sample weight 20:1 (*v*/*w*).

### 3.5. Biomass Cultivation of D. smithii HU04

The selected strain *D. smithii* HU04 was initially inoculated into Z8 medium and cultivated at 25 ± 3 °C temperature with 1500–2000 lux light intensity for 12 h light/12 h dark photoperiod duration in a 1 L flask. The culture was subsequently scaled up to 5 and 20 L transparent flasks under the same conditions until reaching the exponential growth phase. Aeration was provided using filtered air. The cyanobacteria biomass was harvested by centrifugation at 10,000 rpm for 10 min at 4 °C. Biomass was harvested at day 10, triple rinse with distilled water, and drying at 80 °C to constant weight.

Optimized conditions for growth of the strain *D. smithii* HU04. Large-scale culture experiments (150 L flask). Under the optimized conditions—Z8 medium; nitrogen supplied as NaNO_3_ at 150%; phosphorus supplied as KH_2_PO_4_ at 200% of the Z8 standard; 10% inoculum (starter ~10^6^ cells/mL); pH 7.5; continuous aeration; 22–25 °C; 3000–4000 lux—*D. smithii* HU04 reached harvest at day 10. Across the production run, a total of 2.0 kg dried biomass was obtained, providing sample material for extraction, isolation, and bioassays (Table 2).

### 3.6. Extraction and Purification of Compounds

Dried biomass of *D. smithii* HU04 (2.0 kg) was ultrasonically extracted with MeOH at 40 °C three times (each 6.0 L, 3 h). The combined MeOH extracts were evaporated in vacuo to give a dark solid residue (150 g). This crude extract was suspended in water (1.0 L) and partitioned successively with *n*-hexane and CH_2_Cl_2_ to obtain an *n*-hexane fraction (DS1, 15 g), a CH_2_Cl_2_ fraction (DS2, 22 g) and an aqueous layer (DS3). The DS2 fraction was separated on a silica gel column chromatography (CC), eluting with *n*-hexane/acetone (40:1, 20:1, 10:1, 5:1, 1:1, 0:1, *v*/*v*) to give six fractions DS2A (3.2 g), DS2B (2.0 g), DS2C (2.8 g), DS2D (2.5 g), DS2E (3.4 g), and DS2F (4.5 g).

The DS2B fraction was chromatographed on a silica gel CC eluting with *n*-hexane/EtOAc (20/1, *v*/*v*) to give two fractions DS2B1 (700 mg) and DS2B2 (800 mg). The DS2B1 fraction was separated on an RP-18 CC eluting with acetone/water (3/1, *v*/*v*) to give fractions DS2B1A (120 mg) and DS2B1B (172 mg). The DS2B1A fraction was purified by HPLC eluting with 60% acetonitrile (ACN) in water to yield compound **9** (10.0 mg, *t*_R_ 55.5 min). The DS2B2 fraction was fractionated on an RP-18 CC eluting with MeOH/water (1/2, *v*/*v*) to give fractions DS2B2A (350 mg) and DS2B2B (190 mg). The DS2B2A was then purified by HPLC eluting with 30% ACN in water to yield compound **12** (10.0 mg, *t*_R_ 27.2 min).

Next, the DS2D (2.5 g) fraction was chromatographed on a silica gel CC eluting with CH_2_Cl_2_/MeOH (9/1, *v*/*v*) to give two fractions DS2D1 (760 mg) and DS2D2 (450 mg). The DS2D1 fraction was separated on an RP-18 CC eluting with MeOH/water (1/1, *v*/*v*) to give three fractions DS2D1A (100 mg), DS2D1B (185 mg), and DS2D1C (80 mg). The DS2D1B fraction was chromatographed on an HPLC eluting with 20% ACN in water to yield compound **4** (40.0 mg, *t*_R_ 41.6 min). The DS2D2 fraction was fractionated on an RP-18 CC eluting with MeOH/water (1/2, *v*/*v*) to give two fractions DS2D2A (350 mg) and DS2D2B (120 mg). The DS2D2A was then chromatographed on an HPLC eluting with 20% ACN in water to yield compound **5** (60.0 mg, *t*_R_ 32.1 min).

Furthermore, The DS2E fraction was chromatographed on a silica gel CC eluting with CH_2_Cl_2_/MeOH (6/1, *v*/*v*) to give three fractions DS2E1 (500 mg), DS2E2 (600 mg), and DS2E3 (900 mg). The DS2E1 fraction was separated on an RP-18 CC eluting with MeOH/water (1/1.5, *v*/*v*) then purified by HPLC eluting with 27.5% ACN in water to yield compound **2** (8.0 mg, *t*_R_ 40.3 min). The DS2E3 fraction was separated on an RP-18 CC eluting with MeOH/water (1/1.5, *v*/*v*) to yield compound **7** (300 mg).

The aqueous fraction (DS3) was subjected to Diaion HP-20 CC using water to remove sugars and highly polar constituents, followed by 50% and 100% MeOH in water (each 1 L), affording fractions DS3A (18.0 g) and DS3B (10.0 g). The DS3A fraction was chromatographed on a silica gel CC eluting with a CH_2_Cl_2_/MeOH gradient (20/1, 10/1, 5/1, 2.5/1, *v*/*v*) to give four fractions DS3A1 (800 mg), DS3A2 (2.0 g), DS3A3 (10 g), and DS3A4 (4.0 g). The DS3A2 fraction was subjected to a silica gel CC using CH_2_Cl_2_/MeOH/water (5/1/0.1, *v*/*v*/*v*), resulting in DS3A2A (80 mg), DS3A2B (300 mg), and DS3A2C (150 mg). The DS3A2B fraction was purified on an RP-18 CC eluting with MeOH/water (1/1.5, *v*/*v*) then by HPLC eluting with 27.5% ACN in water to yield compound **10** (57.0 mg, *t*_R_ 37.7 min). The DS3A3 fraction was subjected to a silica gel CC using CH_2_Cl_2_/acetone/water (5/1/0.1, *v*/*v*/*v*) to give fractions DS3A3A (300 mg) and DS3A3B (250 mg). The DS3A3A fraction was purified on an RP-18 CC eluting with MeOH/water (1/1.5, *v*/*v*) then by HPLC eluting with 25.0% ACN in water to yield compound **1** (20.0 mg, *t*_R_ 23.2 min). The DS3A3B fraction was purified on an RP-18 CC eluting with MeOH/water (1/3, *v*/*v*) to yield compound **3** (6.0 mg).

The DS3B fraction was chromatographed on a silica gel CC eluting with a CH_2_Cl_2_/MeOH gradient (10/1, 5/1, 2.5/1, *v*/*v*), to give three fractions DS3B1 (750 mg), DS3B2 (1.5 g), and DS3B3 (5.0 g). The DS3B2 fraction was subjected to a silica gel CC using CH_2_Cl_2_/MeOH/water (4/1/0.1, *v*/*v*/*v*) to give fractions DS3B2A (300 mg) and DS3B2B (100 mg). The DS3B2A fraction was purified on an RP-18 CC eluting with MeOH/water (1/1.5, *v*/*v*) to yield compound **11** (40.0 mg). The DS3B3 fraction was subjected to a silica gel CC using CH_2_Cl_2_/acetone/water (1/2/0.1, *v*/*v*/*v*) to give fractions DS3B3A (900 mg) and DS3B3B (400 mg). The DS3B3A fraction was purified on an RP-18 CC eluting with acetone/water (1/1.5, *v*/*v*) then by HPLC eluting with 23% ACN in water to yield compound **6** (40.0 mg, *t*_R_ 47.2 min).

#### 3.6.1. Smithioside A (**1**)

White amorphous powder; ${\left[\alpha \right]}_{D}^{25}$: −36.0 (*c* 0.1, MeOH); C_18_H_34_O_10_, HR-ESI-MS: *m*/*z* 411.2220 [M+H]^+^ (Calcd. for [C_18_H_35_O_10_]^+^, 411.2225); ^1^H- and ^13^C-NMR (CD_3_OD): see Table 3.

#### 3.6.2. Smithioside B (**2**)

White amorphous powder; ${\left[\alpha \right]}_{D}^{25}$: −28.0 (*c* 0.1, MeOH); C_27_H_34_O_14_, HR-ESI-MS: *m*/*z* 581.1855 [M-H]^−^ (Calcd. for [C_27_H_33_O_14_]^−^, 581.1875); ^1^H- and ^13^C-NMR (CD_3_OD): see Table 3.

### 3.7. Acid Hydrolysis

See Supporting Information.

### 3.8. Cytotoxic Assays

Human cancer cell lines, including lung cancer SK-LU-1 and liver cancer (HepG2), were obtained from Milan University, Italy and Long Island University, USA. The cells were maintained and cultured in DMEM supplemented with FBS, trypsin-EDTA, L-glutamine, sodium pyruvate, NaHCO_3_, and penicillin/streptomycin at 37 °C in a humidified atmosphere (5% CO_2_ and 95% air). Cytotoxic effects of compounds were determined using the sulforhodamine B (SRB) assay as previously described [28]. In brief, the cells were incubated with/without compounds for three days in a 96-well culture plate. After incubation, cells were stained with sulforhodamine B and optical density (OD) was measured at 540 nm. The difference in OD between samples and vehicle well during experiments indicated the cell situation induced by the compounds. Results are expressed as the percentage of cell death in comparison with the vehicle as well. The dose–response curves of compounds were generated to determine IC_50_ values of the compounds corresponding to each cell line. Ellipticine was used as a positive control throughout the experiments.

## 4. Conclusions

From the phytoplankton samples collected across six freshwater bodies in Hue city, Vietnam, thirteen microalgal strains were successfully isolated and purified under laboratory conditions. The MeOH extracts of *M. viridis* (HU13) and *D. smithii* (HU04) exhibited significant cytotoxic effects with IC_50_ values of 6.19 ± 0.80 and 4.89 ± 0.76 µg/mL for *M. viridis*, and 9.51 ± 0.84 and 8.32 ± 0.94 µg/mL for *D. smithii* against SK-LU-1 and HepG2 cell lines, respectively. Furthermore, chemical studies of *D. smithii* HU04 led to the isolation of two new compounds, smithioside A (**1**) and smithioside B (**2**) and ten known ones. Compound **12** showed the strongest activity, with IC_50_ values of 9.13 ± 0.89 µM (SK-LU-1) and 7.64 ± 0.46 µM (HepG2). Compounds **5** and **6** exhibited moderate cytotoxic activity on both human cancer cell lines with IC_50_ values ranging from 25.99 to 51.47 µM. Taken together, these findings validate our integrated workflow from strain authentication and culture optimization to dereplication and focused bioassays, an efficient approach for prioritizing freshwater cyanobacterial leads. The findings suggest that *D. smithii* HU04 extracts could be developed for therapeutic purposes targeting cancer.

## Figures and Tables

**Table 1:** List of Collected Microalgae from Various Places of Hue City, Vietnam.

No.	Code	Classification	Place of Collection
		**CYANOBACTERIA**	
		**Chroococcales**	
1	M.a. HU05	*Microcystis aeruginosa*	Nhu Y river
2	M.v. HU13	*Microcystis viridis*	Tinh Tam lake
		**Nostocales**	
3	A.c HU08	*Anabaena circinalis*	Hoa My lake
4	A.f. HU02	*Aphanizomen flos* *-* *aquae*	Huong river
5	D.s. HU04	*Dolichospermum smithii*	Huong river
6	C.b. HU14	*Calothrix braunii*	Tinh Tam lake
7	N.m. HU12	*Nostoc muscorum*	Tinh Tam lake
8	N.p. HU11	*Nostoc punctiforme*	Mung Lake
9	R.r. HU03	*Raphidiopsis raciborskii*	Huong river
		**Oscillatoriales**	
10	L.s HU15	*Lyngbya spiralis*	Tinh Tam lake
11	P.s HU16	*Phormidium stagnina*	Mung lake
12	P.s. HU06	*Planktothrix* *subtilis*	Nhu Y river
		**CHLOROPHYTA**	
13	S.q. HU07	*Scenedesmus quadricauda*	Hoa My lake

**Table 2:** Biomass Information of Microalgae and Cytotoxic effects.

No.	Microalgae Name	Culture Medium	Dried Biomass (g/flask)	MeOH Extract (g)	Extraction Yield (%)	IC_50_ (µg/mL)	
						SK-LU-1	HepG2
1	*Microcystis aeruginosa*	Z8	3.3	0.43	13.03	78.93 ± 7.42	47.39 ± 6.53
2	*Microcystis viridis*	Z8	3.1	0.35	11.29	6.19 ± 0.80	4.89 ± 0.76
3	*Anabaena circinalis*	Z8	3.0	0.37	12.33	88.55 ± 6.27	72.82 ± 3.99
4	*Aphanizomen flos* *-* *aquae*	Z8	1.7	0.21	12.36	>100	>100
5	*Dolichospermum smithii*	Z8	3.9	0.42	10.77	9.51 ± 0.84	8.32 ± 0.94
6	*Calothrix braunii*	Z8	2.1	0.28	13.33	>100	>100
7	*Nostoc muscorum*	Z8	2.9	0.36	12.41	>100	>100
8	*Nostoc punctiforme*	Z8	3.0	0.46	15.33	>100	>100
9	*Raphidiopsis raciborskii*	Z8	3.0	0.31	10.33	>100	>100
10	*Lyngbya spiralis*	Z8	1.8	0.22	12.22	>100	>100
11	*Phormidium stagnina*	Z8	1.5	0.20	13.33	>100	>100
12	*Planktothrix* *subtilis*	Z8	4.1	0.53	12.93	>100	>100
13	*Scenedesmus quadricauda*	BBM	4.2	0.52	12.62	>100	>100

**Table 3:** NMR Data for Compounds **1** and **2** in CD_3_OD.

C		1			2
	*δ*_C_^a^	*δ*_H_ ^b^ (Mult., *J* = Hz)		*δ*_C_^a^	*δ*_H_ ^b^ (Mult., *J* = Hz)
1	19.6	1.18 (d, 6.0)		138.9	-
2	75.3	3.90 (m)		129.2	7.34 (d, 7.8)
3	38.4	1.45 (m)/1.61 (m)		129.2	7.22 (t, 7.8)
4	26.1	1.39 (m)		128.6	7.17 (t, 7.8)
5	33.1	1.31 (m)		129.2	7.22 (t, 7.8)
6	23.7	1.34 (m)		129.2	7.34 (d, 7.8)
7	14.4	0.93 (t, 6.6)		71.9	4.55 (d, 11.4) 4.91 (d, 11.4)
**6-*O-*Glc**					
1′	100.7	4.41 (d, 7.8)		102.2	4.44 (d, 7.8)
2′	78.6	3.33 (dd, 7.8, 9.0)		78.9	3.50 (dd, 7.8, 9.6)
3′	78.8	3.49 (t, 9.0)		78.6	3.48 (m)
4′	71.9	3.31 (t, 9.0)		71.8	3.32 (m)
5′	77.7	3.24 (m)		78.0	3.27 (m)
6′	62.9	3.68 (dd, 5.4, 12.0) 3.86 (dd, 2.4, 12.0)		62.8	3.70 (dd, 6.0, 12.0) 3.90 (dd, 2.4, 12.0)
**2′-*O*-Api**					
1″	110.5	5.41 (d, 1.8)		110.4	5.44 (d, 1.2)
2″	78.0	3.95 (d, 1.8)		78.5	4.01 (d, 1.2)
3″	80.8	-		79.2	-
4″	75.4	3.73 (d, 9.6) 4.08 (d, 9.6)		75.4	3.72 (d, 9.6) 4.04 (d, 9.6)
5″	66.3	3.62 (d, 11.4) 3.66 (d, 11.4)		66.8	4.27 (d, 11.4) 4.40 (d, 11.4)
**5″-*O*-** **Syr**					
1‴				121.0	-
2‴, 6‴				108.6	7.36 (s)
3‴, 5‴				149.1	-
4‴				143.0	-
7‴				168.0	-
3‴/5‴-OMe				57.0	3.90 (s)

^a^ 150 MHz, ^b^ 600 MHz, Glc, glucopyranosyl, Api, apiofuranosyl, Syr, syringyl.

**Table 4:** Effects of Compounds **1**–**12** from *D. smithii* on the Growth of Human Cancer Cells.

Compound	IC_50_ (µM)	
	SK-LU-1	HepG2
**1**	>100	>100
**2**	>100	>100
**3**	>100	>100
**4**	79.80 ± 1.34	67.53 ± 2.87
**5**	51.47 ± 4.31	44.81 ± 1.32
**6**	29.77 ± 1.56	25.99 ± 2.17
**7**	>100	>100
**8**	>100	>100
**9**	84.06 ± 6.53	67.12 ± 5.91
**10**	74.69 ± 3.54	61.05 ± 3.50
**11**	>100	>100
**12**	9.13 ± 0.89	7.64 ± 0.46
**Ellipticine**	3.51 ± 0.38	3.42 ± 0.45

Ellipticine was used as a positive control.

## Data Availability

Data are contained within the article and the Supplementary Materials.

## Supplementary Materials

The following supporting information can be downloaded at: https://www.mdpi.com/article/10.3390/molecules31010165/s1, Table S1: Cell density of *D. smithii* HU04 at different nitrogen levels; Table S2: Cell Density of *D. smithii* HU04 at Different Phosphorus Levels; Figure S1: Growth of *D. smithii* HU04 at Different Initial Inoculum Density; Figure S2: Growth of *D. smithii* HU04 at Different Nitrogen Levels in Z8 Medium; Figure S3: Growth of *D. smithii* HU04 at Different Phosphorus Levels in Z8 Medium; Figure S4: Photographs of Laboratory-Scale Cultivation of *D. smithii*; Figure S5: Photographs of *D. smithii* Harvest; Figure S6: HR-ESI-MS Spectrum of Compound **1**; Figure S7: ^1^H-NMR Spectrum of Compound **1**; Figure S8: ^1^H-NMR Spectrum of Compound **1** (Expanded); Figure S9: ^13^C-NMR Spectrum of Compound **1**; Figure S10: HSQC Spectrum of Compound **1**; Figure S11: HMBC Spectrum of Compound **1**; Figure S12: COSY Spectrum of Compound **1**; Figure S13. GC Chromatogram of TMS-Sugar Derivatives for Compound **1**; Figure S14: HR-ESI-MS Spectrum of Compound **2**; Figure S15: ^1^H-NMR Spectrum of Compound **2**; Figure S16: 1H-NMR Spectrum of Compound **2** (Expanded); Figure S17: ^13^C-NMR Spectrum of Compound **2**; Figure S18: ^13^C-NMR Spectrum of Compound **2** (Expanded); Figure S19: HSQC Spectrum of Compound **2**; Figure S20: HMBC Spectrum of Compound **2**; Figure S21: COSY Spectrum of Compound **2**.
