# Supplementary material for: Screening of Bioactive Microalgae from Freshwaters, Collected in Hue, Vietnam: Cytotoxic Constituents from Dolichospermum smithii HU04
Source: Molecules. 2026 Jan 1;31(1):165. doi: 10.3390/molecules31010165 (PMC12787843; doi:10.3390/molecules31010165)
Supplement: Supplementary file 1 [file molecules-31-00165-s001.zip › molecules-4010057-supplementary.pdf]

Supporting Information

Screening of Bioactive Microalgae from Freshwaters, collected in Hue, Vietnam: Cytotoxic Constituents from *Dolichospermum smithii* HU04

Nguyen Thi Minh Hang [1], Nguyen Thi Thu Ha [1], Hoang Duc Manh [2], Duong Thi Thuy [3], Hoang Thi Quynh [3], Nguyen Thi Thu Lien [4], Nguyen Thi Tu Oanh [1], Tran Huu Giap [1], Buu Huu Tai [1], Doan Thi Mai Huong [1], Ngo Quoc Anh [1], and Nguyen Xuan Nhiem [1,5,*]

[1] Institute of Chemistry, Vietnam Academy of Science and Technology (VAST), 18 Hoang Quoc Viet, Nghia Do, Hanoi 10072, Vietnam
[2] National Foundation for Science and Technology Development, Ministry of Science and Technoogy, 113 Tran Duy Hung, Yen Hoa, Hanoi, Vietnam
[3] Institute of Science and Technology for Energy and Environment, VAST, 18 Hoang Quoc Viet, Nghia Do, Hanoi 10072, Vietnam
[4] Institute of Applied Research for Science and Technology, University of Sciences, Hue 530000, Vietnam
[5] Graduate University of Science and Technology, VAST, 18 Hoang Quoc Viet, Nghia Do, Hanoi 10072, Vietnam

[1*] Correspondence: nxnhiem@yahoo.com (N.X.N); Tel.+84-24-3791-9977

Table of Contents

3.5.1. Effect of Initial Inoculum Density on the Growth of *Dolichospermum smithii* HU04 1
3.5.2. Effects of Media Formulations on the Growth of *D. smithii* HU04 1
3.5.2.1. Nitrogen Level ($NaNO_3$) 1
3.5.2.2. Phosphorus Level (KH_2PO_4) 1
3.3. Large-Scale Cultivation of D. smithii HU04 1
3.7. Acid Hydrolysis 2
Table S1. *Cell density of D. smithii HU04 at different nitrogen levels* 3
Table S2. *Cell Density of D. smithii HU04 at Different Phosphorus Levels.* 3
Figure S1. *Growth of D. smithii HU04 at Different Initial Inoculum Density.* 4
Figure S2. *Growth of D. smithii HU04 at Different Nitrogen Levels in Z8 Medium.* 4
Figure S3. *Growth of D. smithii HU04 at Different Phosphorus Levels in Z8 Medium.* 5
Figure S4. *Photographs of Laboratory-Scale Cultivation of D. smithii.* 5
Figure S5. *Photographs of D. smithii Harvest* 5
Figure S6. *HR-ESI-MS Spectrum of Compound **1*** 6
Figure S7*. ^{1}H-NMR Spectrum of Compound **1*** 6
Figure S8. *^{1}H-NMR Spectrum of Compound **1** (Expanded)* 7
Figure S9. *^{13}C-NMR Spectrum of Compound **1*** 7
Figure S10. *HSQC Spectrum of Compound **1*** 8
Figure S11. *HMBC Spectrum of Compound **1*** 8
Figure S12. *COSY Spectrum of Compound **1*** 9
Figure S13. *GC Chromatography of TMS-Sugar Derivatives for Compound **1*** 9
Figure S14. *HR-ESI-MS Spectrum of Compound **2*** 10
Figure S15. *^{1}H-NMR Spectrum of Compound **2*** 10
Figure S16*. ^{1}H-NMR Spectrum of Compound **2** (Expanded)* 11
Figure S17. *^{13}C-NMR Spectrum of Compound **2*** 11
Figure S18. *^{13}C-NMR Spectrum of Compound **2** (Expanded)* 12
Figure S19. *HSQC Spectrum of Compound **2*** 12
Figure S20. *HMBC Spectrum of Compound **2*** 13
Figure S21. *COSY Spectrum of Compound **2*** 13

Material and Methods

3.5.1. Effect of Initial Inoculum Density on the Growth of *Dolichospermum smithii* HU04

In microalgal batch cultivation, initial inoculum density is a key determinant of growth kinetics and time to maximum cell density. Here, upon scaling cultivation from 100 mL to 5 L and 20 L flask, starting volume fractions of 5, 10, 15, and 20% were tested using a seed culture of approximately 0.5×10^5 cells/mL. Growth of *D. smithii* H04 was monitored, and the results are shown in Figure S1. From Figure S1, the 20% inoculum yielded the earliest biomass maximum after 9 days, reaching 1.24×10^6 cells/mL. The highest maximum was obtained with the 10% inoculum (1.41×10^6 cells/mL on day 11). The lowest maximum occurred with the 5% inoculum (0.986×10^6 cells/mL on day 12), and the peak appeared not yet stabilized. Thus, higher final maxima tended to require longer cultivation times; lower initial densities prolonged time-to-peak.

3.5.2. Effects of Media Formulations on the Growth of *D. smithii* HU04

When scaling up cell production, biomass development typically changes. In addition to adjusting the initial inoculum density, concentrations of the essential nutrients nitrogen (N) and phosphorus (P) in Z8 medium were varied to identify conditions that maximize biomass for downstream studies.

3.5.2.1. Nitrogen Level ($NaNO_3$)

Along with light and pH, nitrogen is a primary nutritional factor affecting microalgal growth. Under nutrient-limited culture, increasing nutrient concentrations can influence algal growth. To identify an optimal media for rapid, high biomass production, growth of *D. smithii* D.s.H04 was evaluated in Z8 medium containing 0, 50, 100, 150, and 200% of the standard $NaNO_3$. Growth was strongly dependent on nitrogen level. In general, as nitrogen increased from 0, 50, 100 and to 150%, growth improved; at 200% nitrogen growth declined relative to 150%. The maximum growth was obtained at 150% N (Table S1, Figure S2).

3.5.2.2. Phosphorus Level (KH_2PO_4)

Phosphorus is another limiting factor in microalgal cultivation. For *D. smithii* HU04, decreasing KH_2PO_4 reduced growth, whereas elevating KH_2PO_4 increased growth in a concentration-dependent manner. Specifically, the deficient media 0 and 50% P markedly suppressed growth: at the typical peak time (day 10), cell density dropped from 25.37×10^4 cells/mL (100% P) to 13.68×10^4 (50% P) and 7.32×10^4 (0% P). Conversely, 150 and 200% P further improved growth; 200% P gave the highest values around day 10 (Table S2, Figure S3).

3.3. Large-Scale Cultivation of D. smithii HU04

Based on the optimization above, biomass cultivation of the cyanobacterium *Dolichospermum smithii* HU04 was performed at laboratory scale in 150 L flask under the following conditions:

Medium: Z8

Nitrogen source: $NaNO_3$ at 150% of standard Z8
Phosphorus source: KH_2PO_4 at 200% of standard Z8
Phosphorus source: KH_2PO_4 at 200% of standard Z8
Inoculum density: 10% (seed culture ~10^6 cells/mL)
pH: 7.5
Aeration: continuous
Temperature: 22–25 °C
Illumination: 3000–4000 lux
Harvest time: day 10
When cultures reached approximately 27.65 × 10^4 cells mL^{-1} (~day 10; early stationary phase), aeration was stopped and cultures were allowed to settle for 3–4 h. Supernatant was siphoned off; biomass was collected by bag filtration to obtain a slurry (about 5–10% of the original culture volume). The slurry was centrifuged at 3,000 rpm for 10 min, washed with distilled water 3×, and dried at 80 °C to constant weight.

3.7. Acid Hydrolysis

Each compound **1** and **2** (2.0 mg) was dissolved in 1.0 N HCl (dioxane–H_2O, 1:1, v/v, 1.0 mL) and then heated to 80°C in a water bath for 3 h. The acidic solution was neutralized with silver carbonate and the solvent thoroughly driven out under N_2 gas overnight. After extraction with $CHCl_3$, the aqueous layer was concentrated to dryness using N_2 gas. The residue was dissolved in 0.1 ml of dry pyridine, and then L-cysteine methyl ester hydrochloride in pyridine (0.06 M, 0.1 ml) was added to the solution. The reaction mixture was heated at 60 °C for 2 h, and 0.1 ml of trimethylsilylimidazole solution was added, followed by heating at 60 °C for 1.5 h. The dried product was partitioned with *n*-hexane and H_2O (0.1 ml, each), and the organic layer was analyzed by GC: Column: column of SPB-1 (0.25 mm × 30 m); detector FID, column temp 212 °C, injector temp 270 °C, detector temp 300 °C, carrier gas He (2.0 ml/min). The retention times of persilylated glucose and apiose were founded to be 13.62 and 6.20 min, respectively, when compared with the standard solutions prepared by the same reaction from the standard monosaccharides. The retention times of persilylated D-glucose, L-glucose, D-apiose, and L-apiose rhamnose were 13.62, 13.87, 6.20 and 6.73 min, respectively.

Table S1. Cell density of *D. smithii* HU04 at different nitrogen levels.

Media	Day 0	Day 2	Day 4	Day 6	Day 8	Day 10	Day 12	Day 14
**0% N**	3.26	3.92	4.39	5.38	6.47	7.04	6.56	5.37
**50% N**	3.27	5.03	6.63	9.47	10.01	13.14	15.10	11.00
**150% N**	3.32	6.45	10.95	18.47	24.58	27.37	32.46	31.47
**200% N**	3.10	6.37	10.34	15.36	17.76	25.16	24.36	22.13
**100% N**	3.42	5.34	14.25	17.55	20.15	25.37	22.36	19.18

Cell density (×10^4 cells/mL)

Table S2. Cell Density of *D. smithii* HU04 at Different Phosphorus Levels.

Medium	Day 0	Day 2	Day 4	Day 6	Day 8	Day 10	Day 12	Day 14
**100% P**	3.26	5.34	14.25	17.55	20.15	25.37	22.36	19.18
**200% P**	3.43	6.01	16.45	19.98	24.36	27.69	25.56	21.36
**150% P**	3.37	5.78	15.37	18.25	22.36	26.59	23.12	21.12
**50% P**	3.15	4.90	5.86	7.57	9.37	13.68	11.76	9.94
**0% P**	3.04	3.50	4.16	5.17	6.34	7.32	6.65	4.36

Cell density (×10^4 cells/mL)

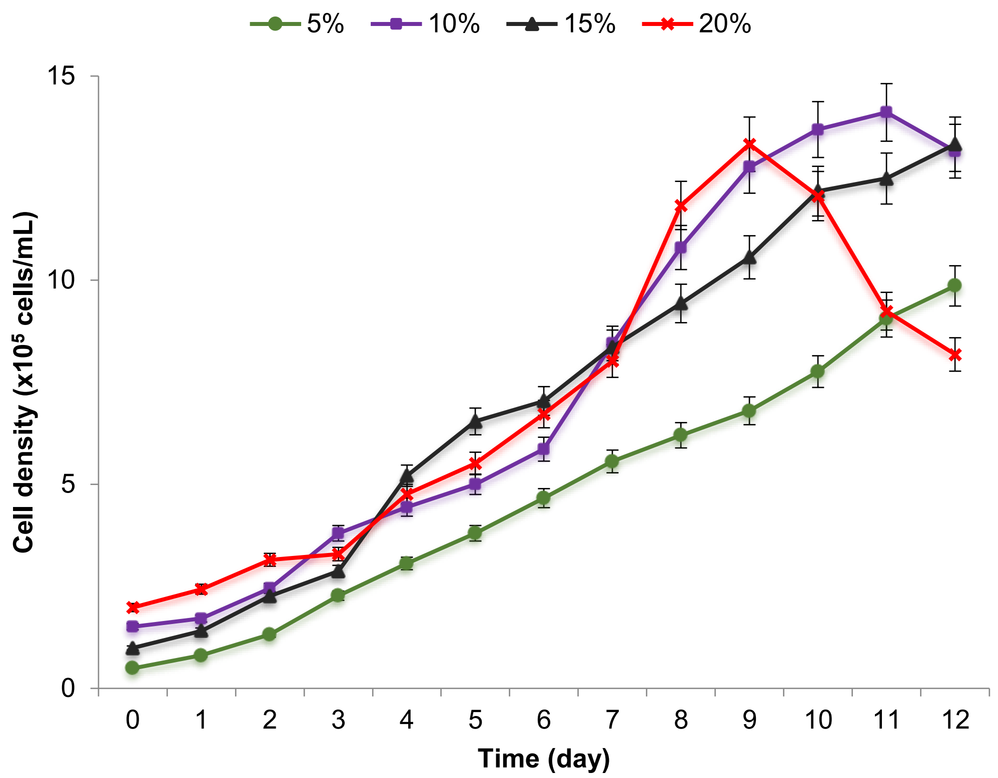

Figure S1. Growth of *D. smithii* HU04 at Different Initial Inoculum Density.

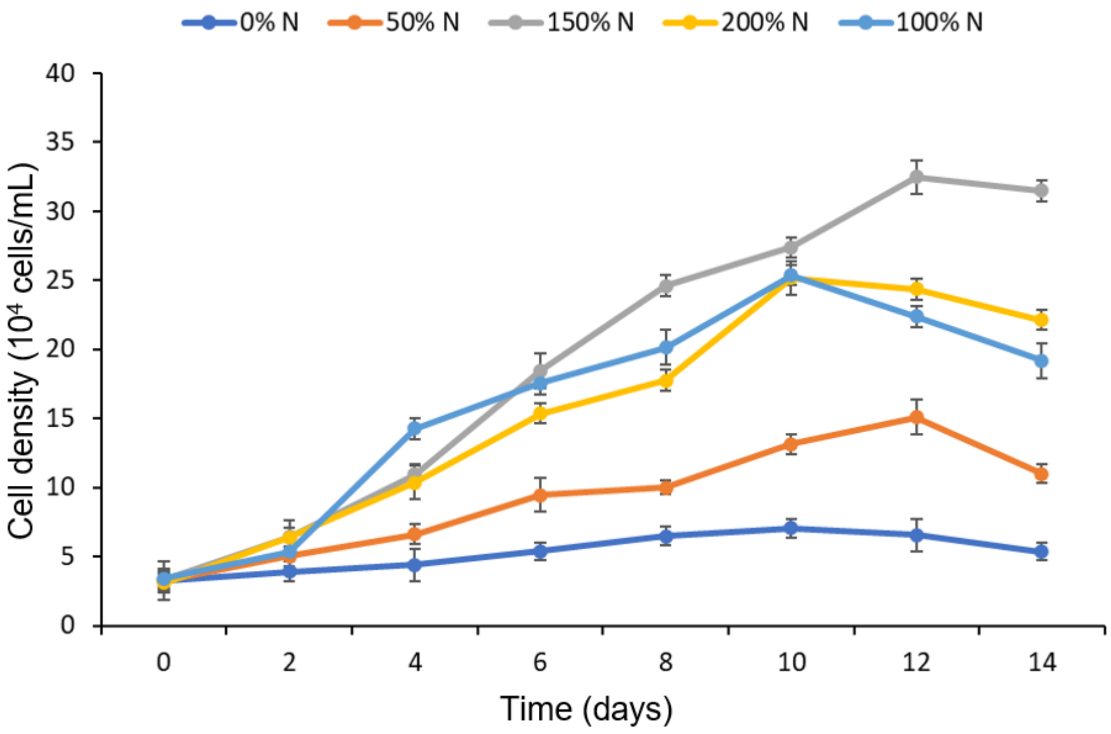

Figure S2. Growth of *D. smithii* HU04 at Different Nitrogen Levels in Z8 Medium.

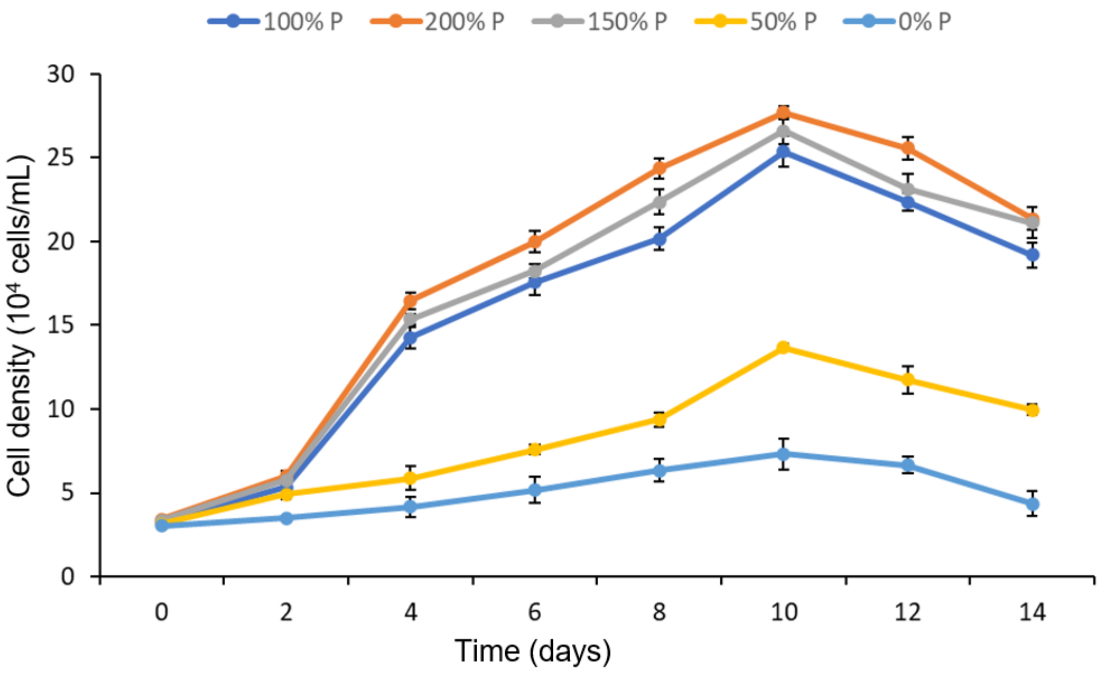

Figure S3. Growth of *D. smithii* HU04 at Different Phosphorus Levels in Z8 Medium.

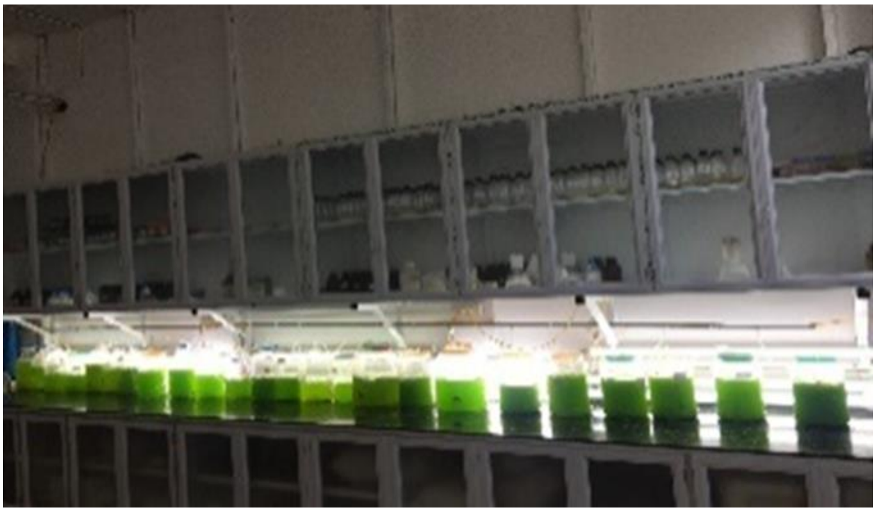

Figure S4. Photographs of Laboratory-Scale Cultivation of *D. smithii*.

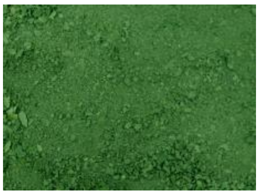

Figure S5. Photographs of *D. smithii* Harvest

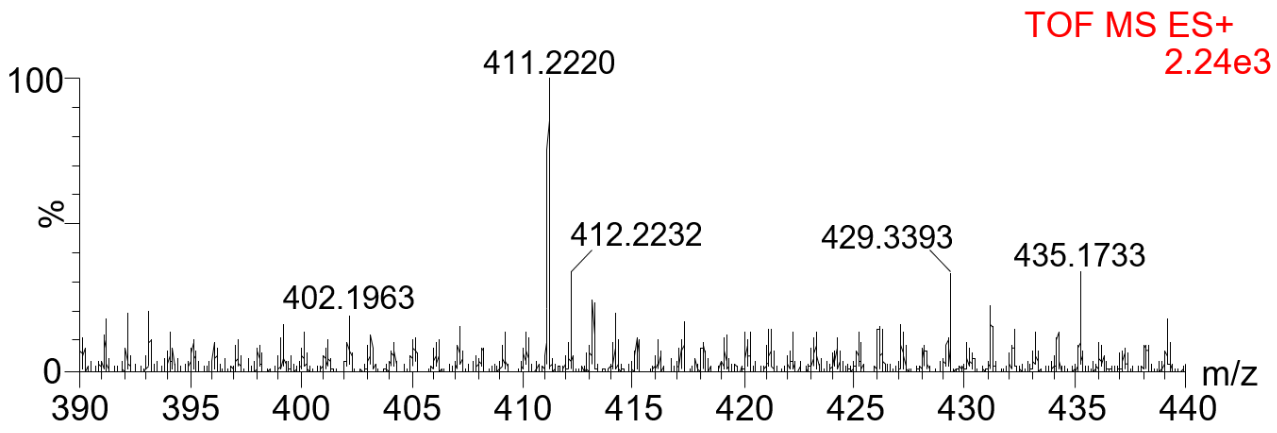

Figure S6. HR-ESI-MS Spectrum of Compound **1**

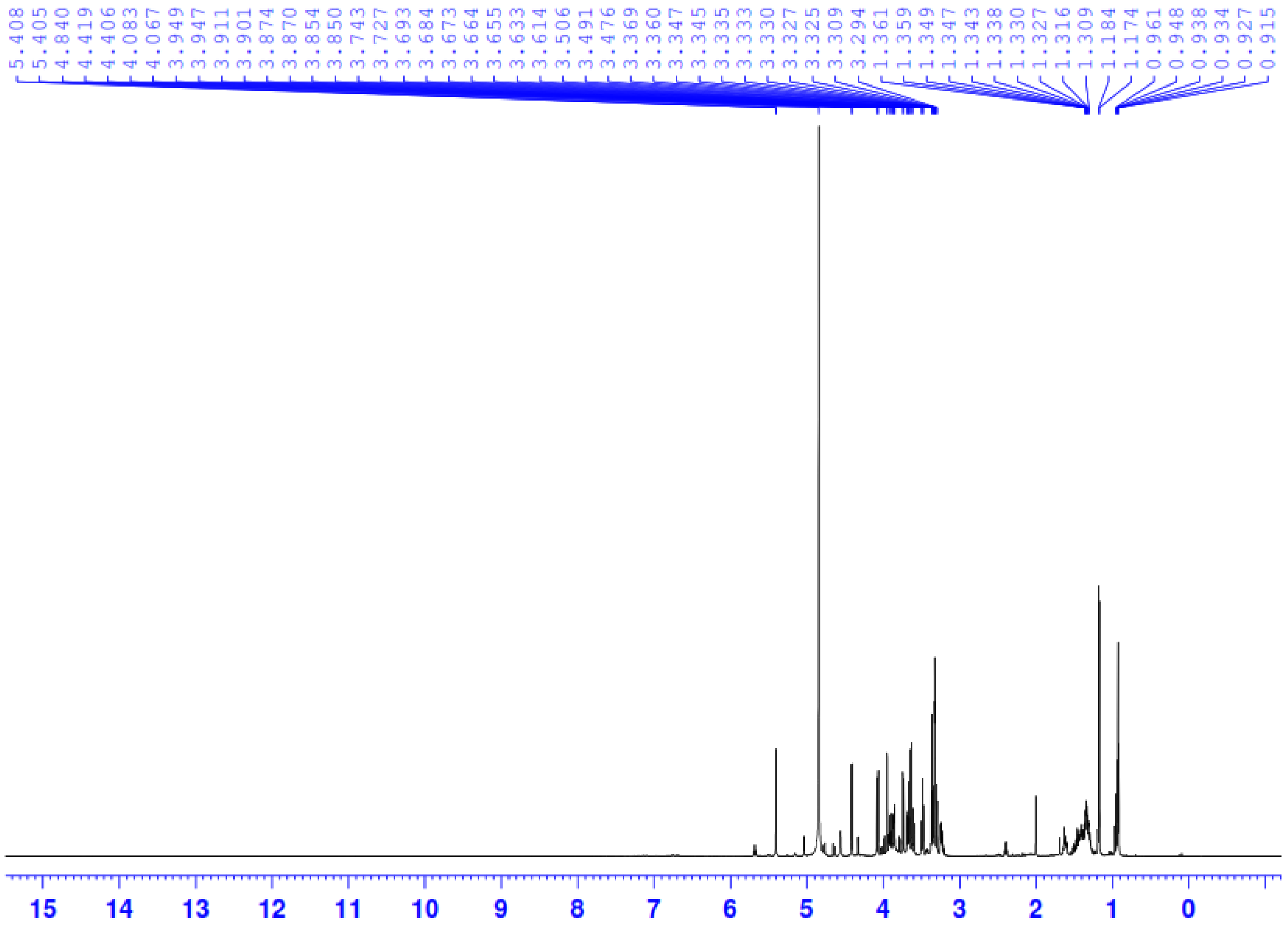

Figure S7. ^{1}H-NMR Spectrum of Compound **1**

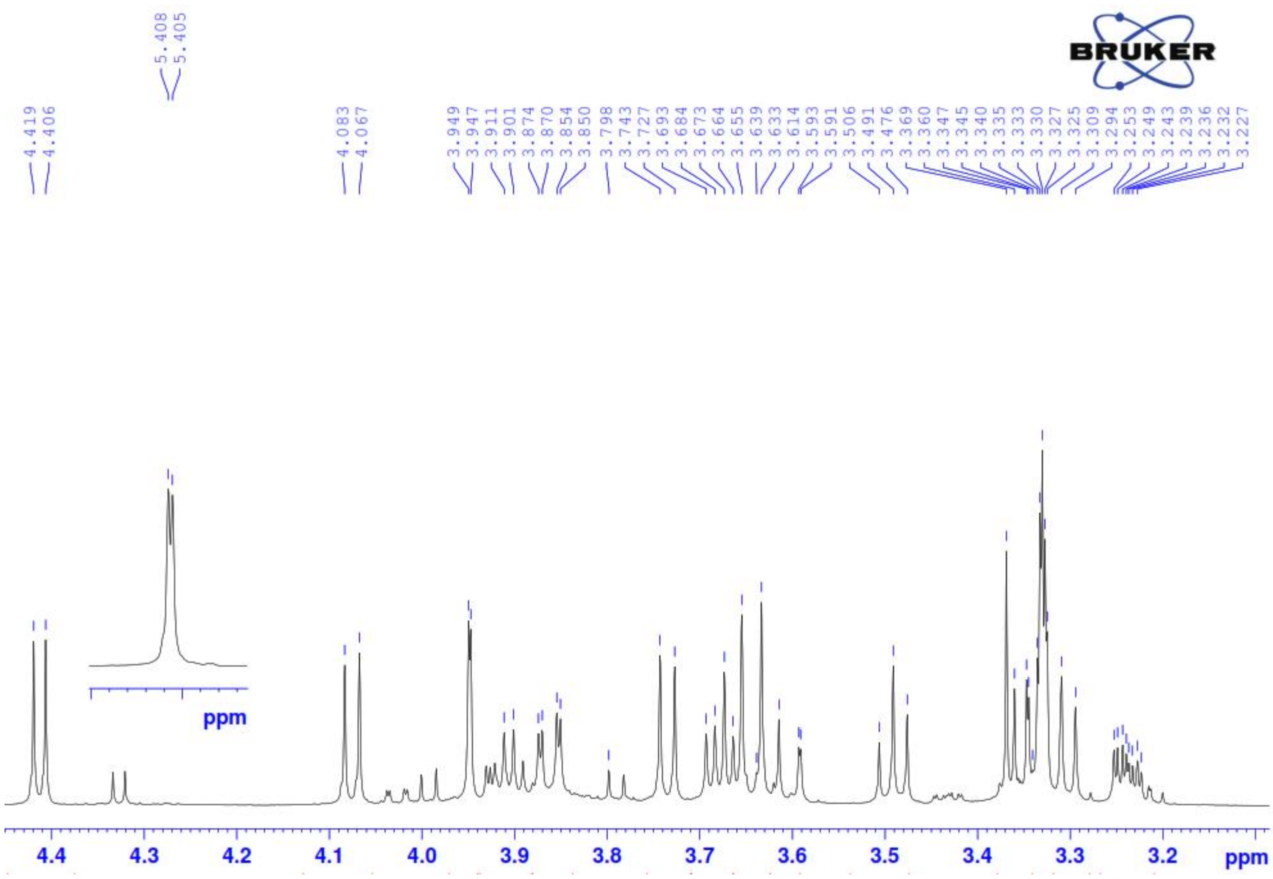

Figure S8. ¹H-NMR Spectrum of Compound **1** (Expanded)

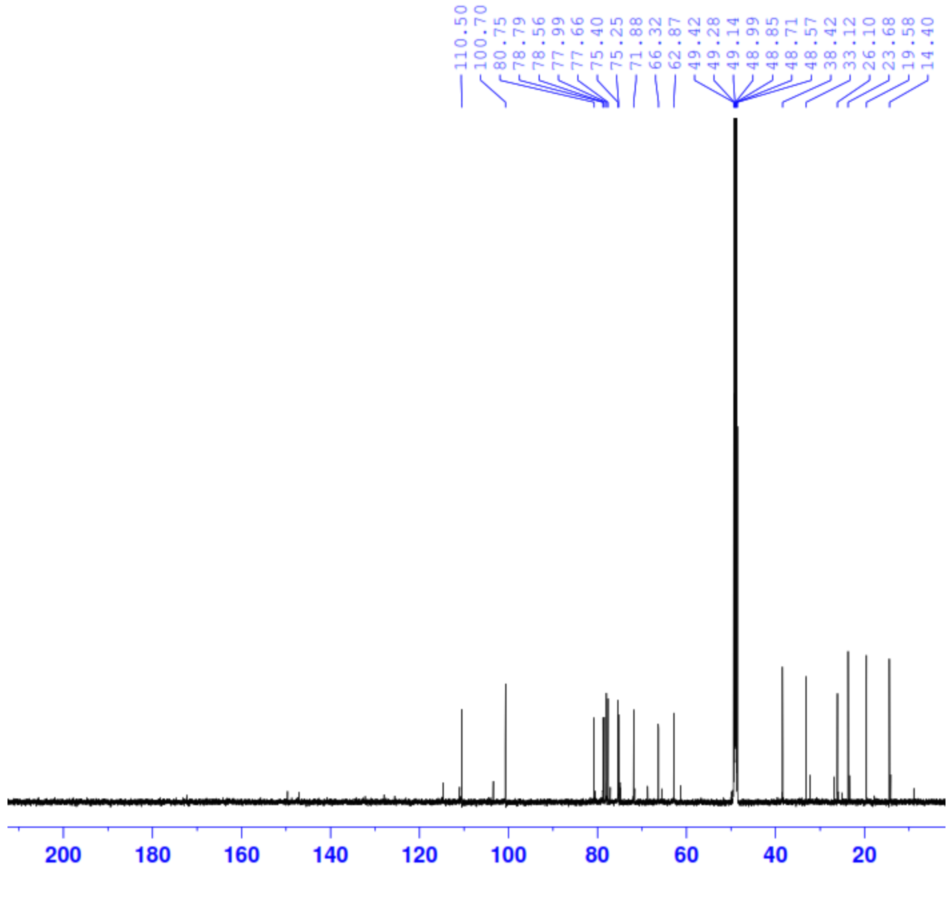

Figure S9. ^{13}C-NMR Spectrum of Compound **1**

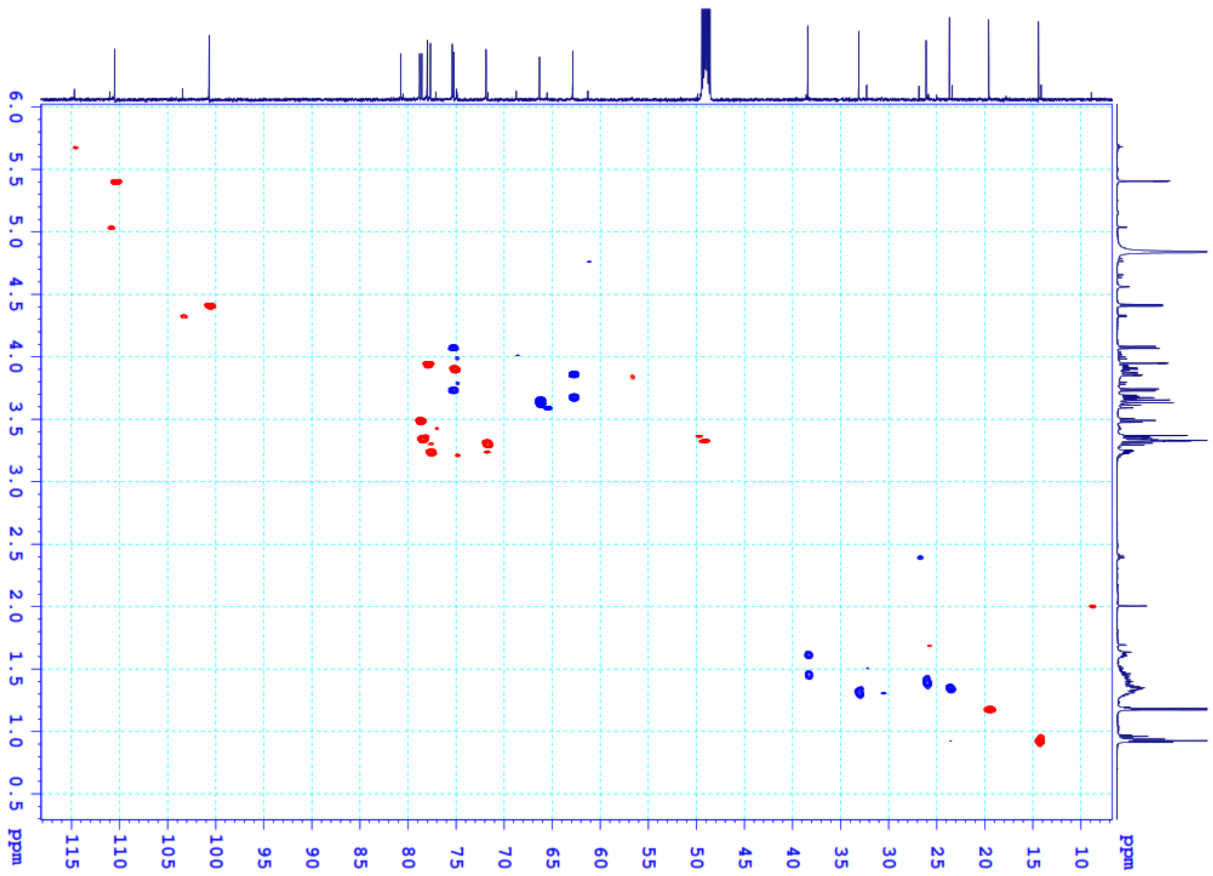

Figure S10. HSQC Spectrum of Compound **1**

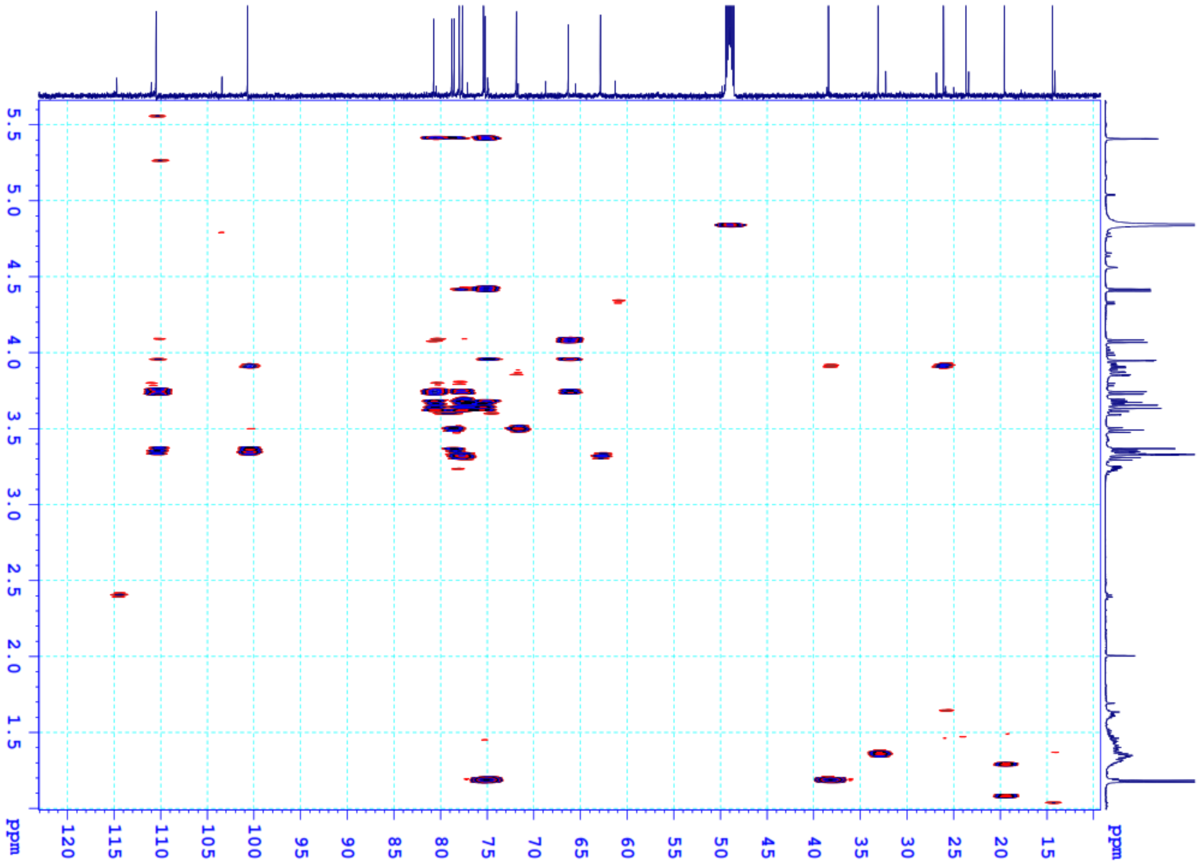

Figure S11. HMBC Spectrum of Compound **1**

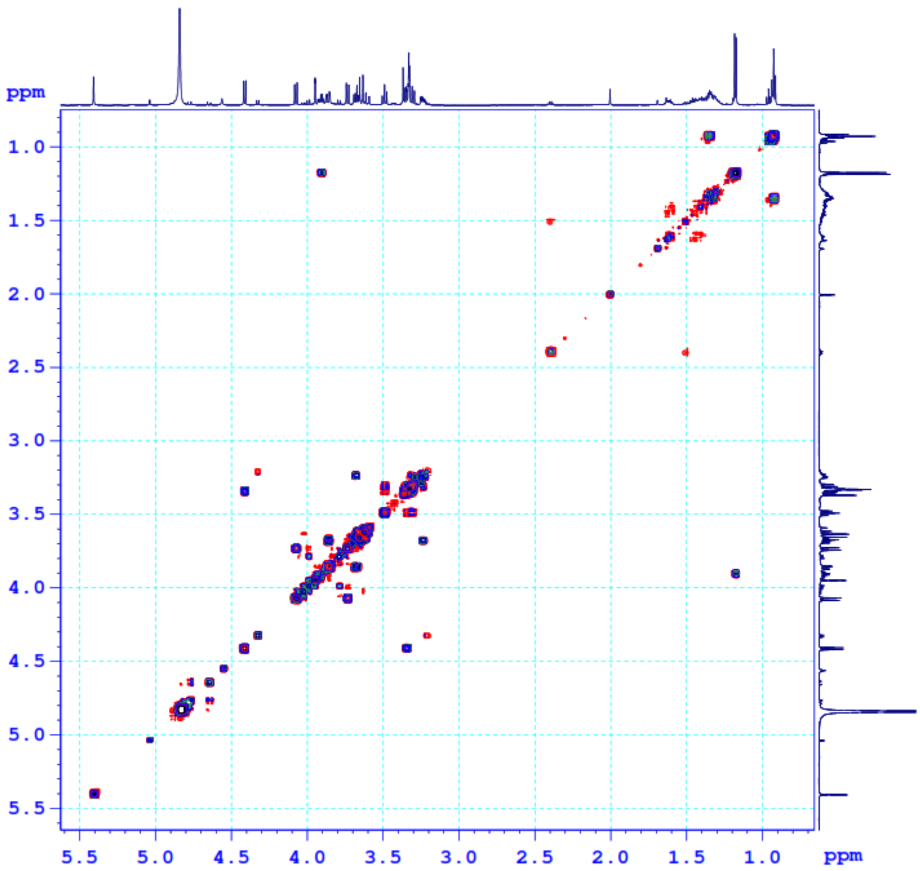

Figure S12. COSY Spectrum of Compound **1**

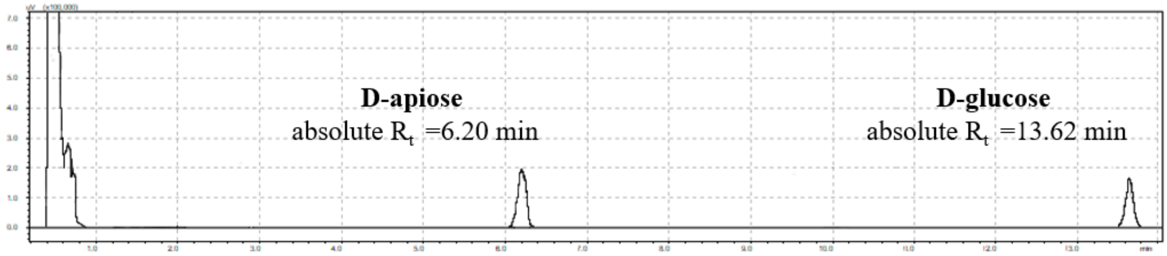

Figure S13. GC Chromatography of TMS-Sugar Derivatives for Compound **1**

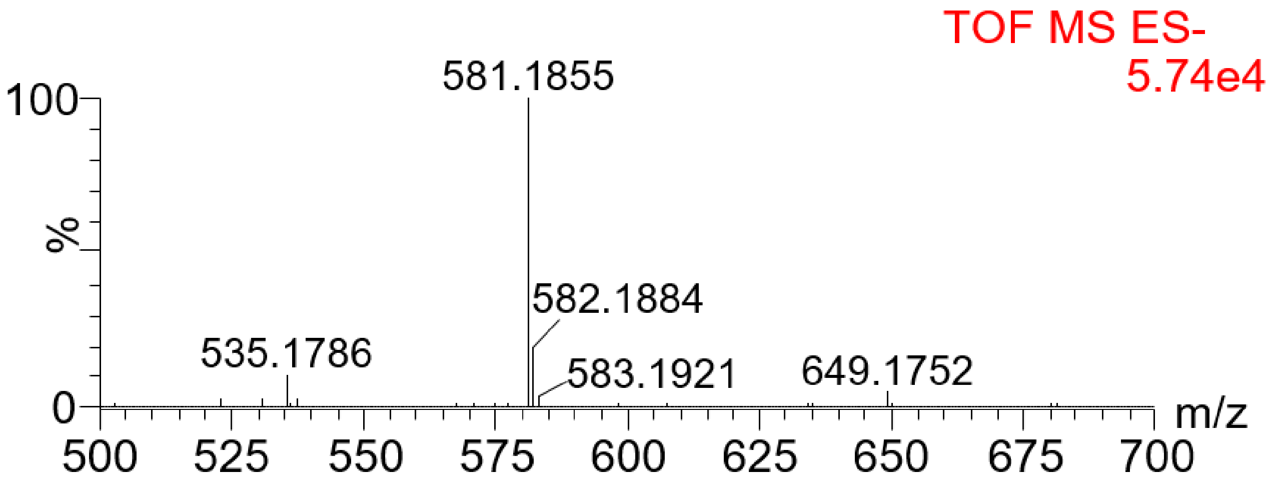

Figure S14. HR-ESI-MS Spectrum of Compound **2**

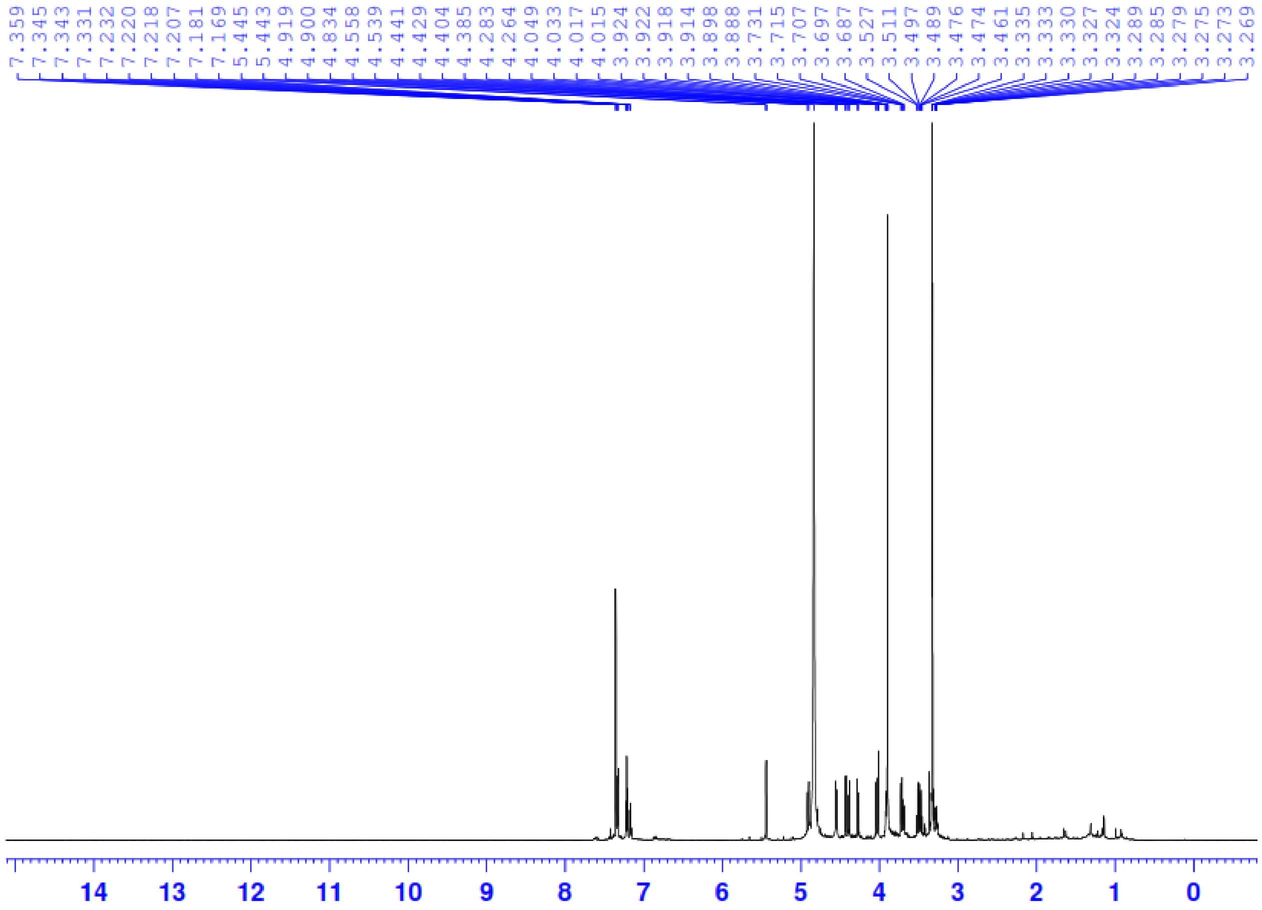

Figure S15. ^{1}H-NMR Spectrum of Compound **2**

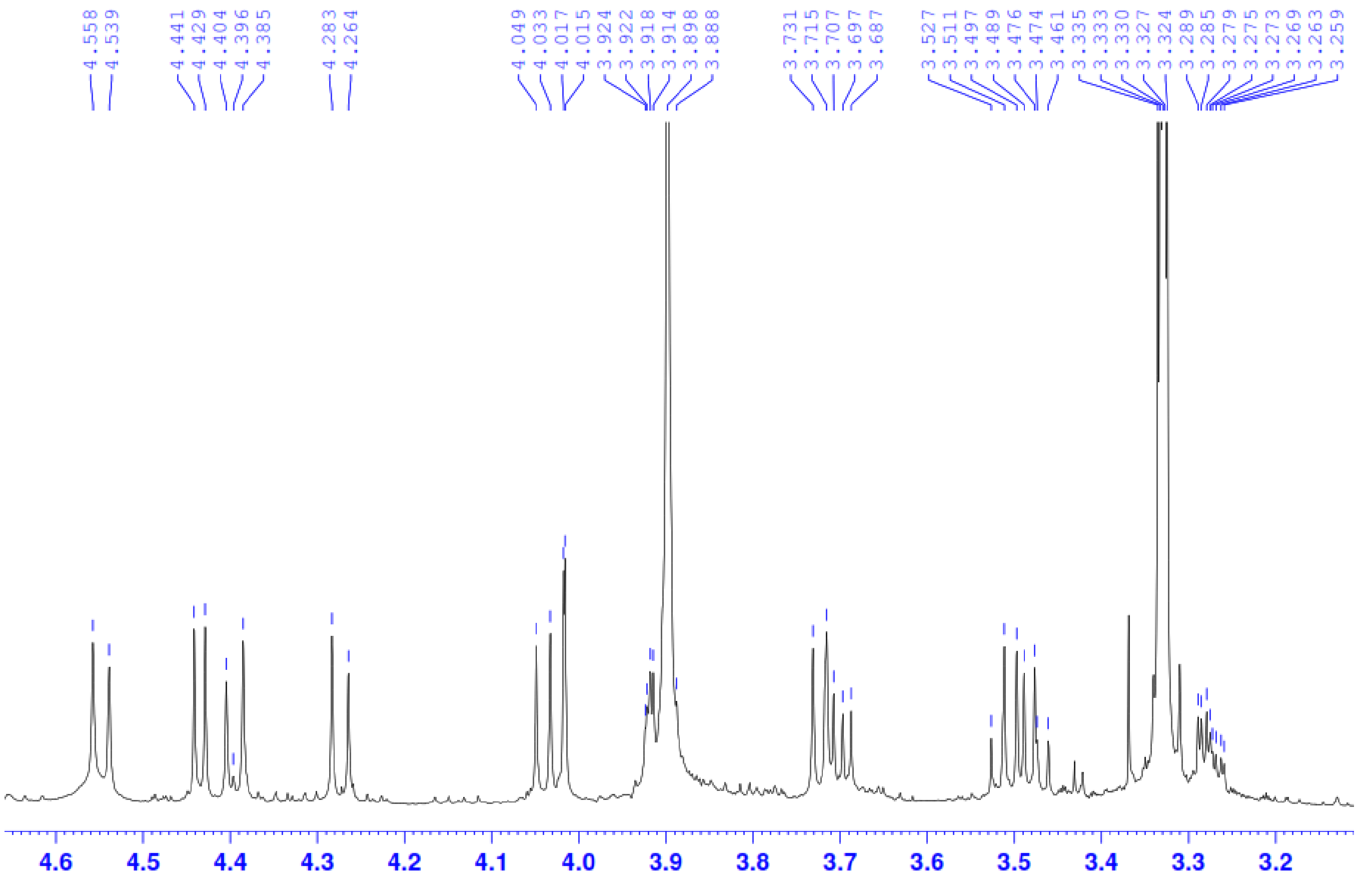

Figure S16. ^{1}H-NMR Spectrum of Compound **2** (Expanded)

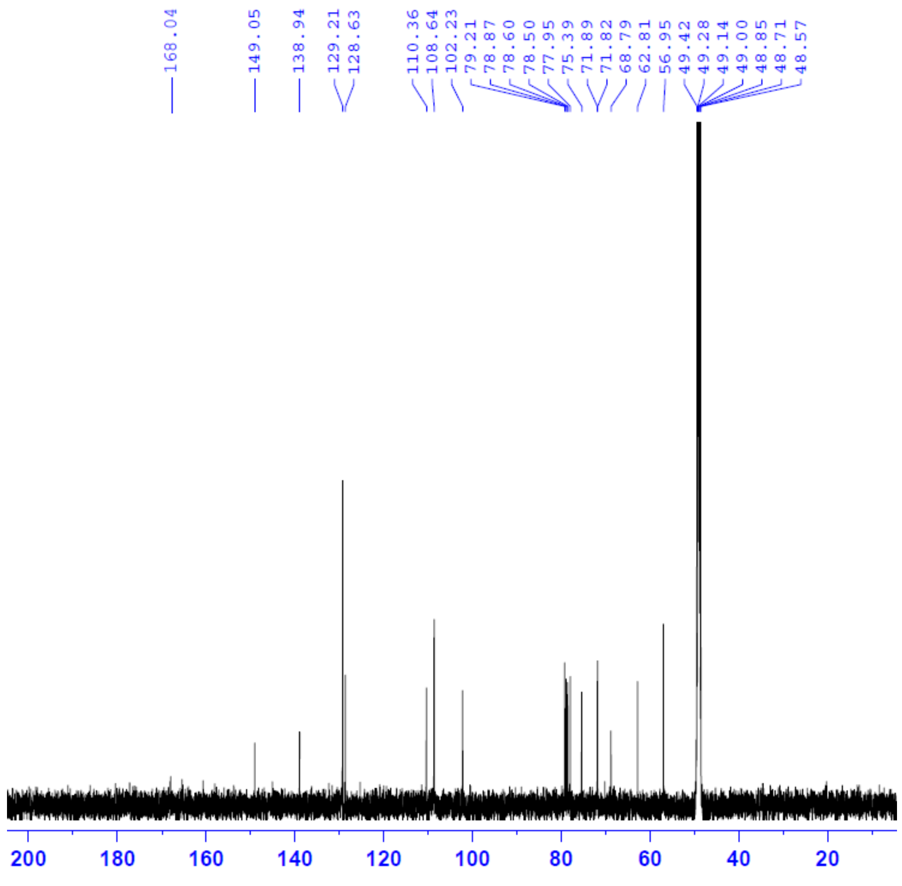

Figure S17. ^{13}C-NMR Spectrum of Compound **2**

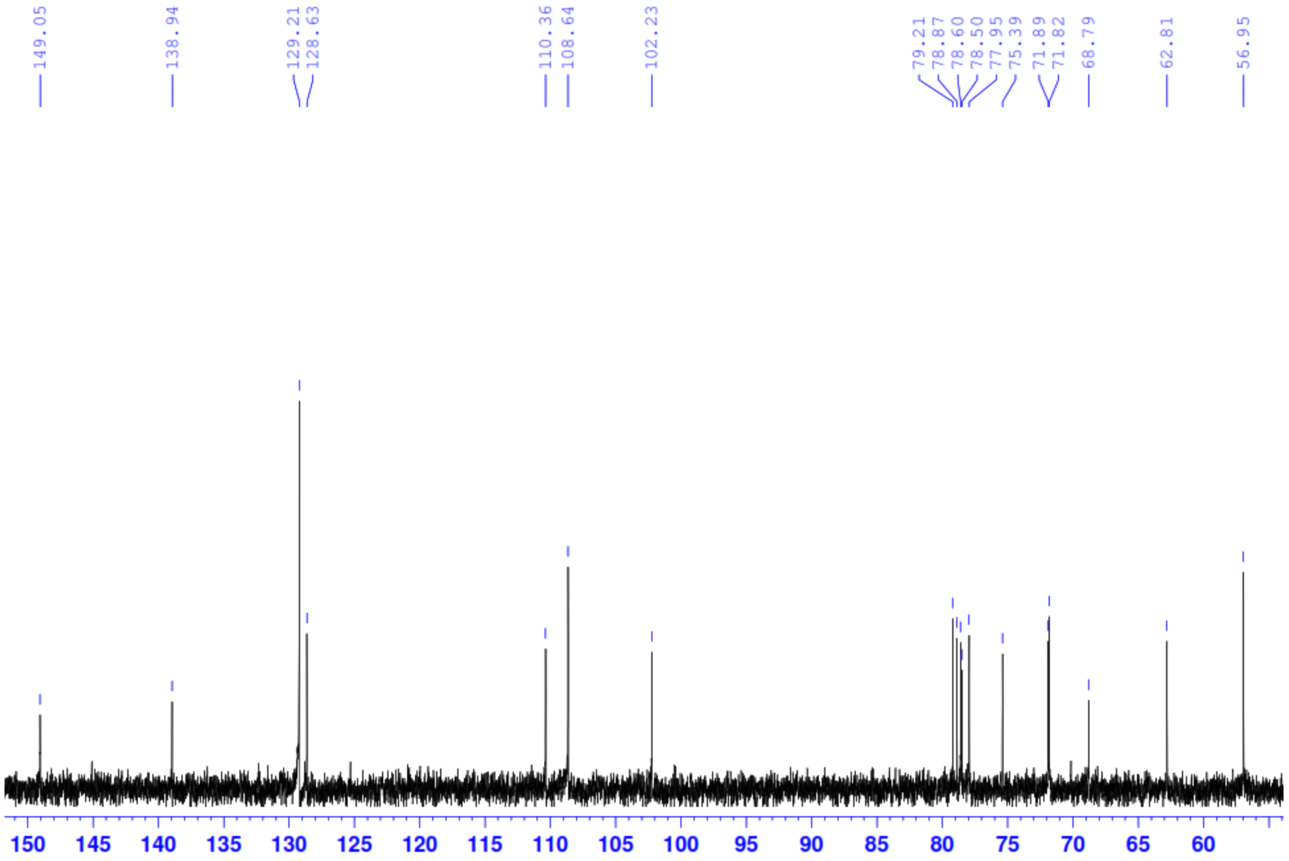

Figure S18. ^{13}C-NMR Spectrum of Compound **2** (Expanded)

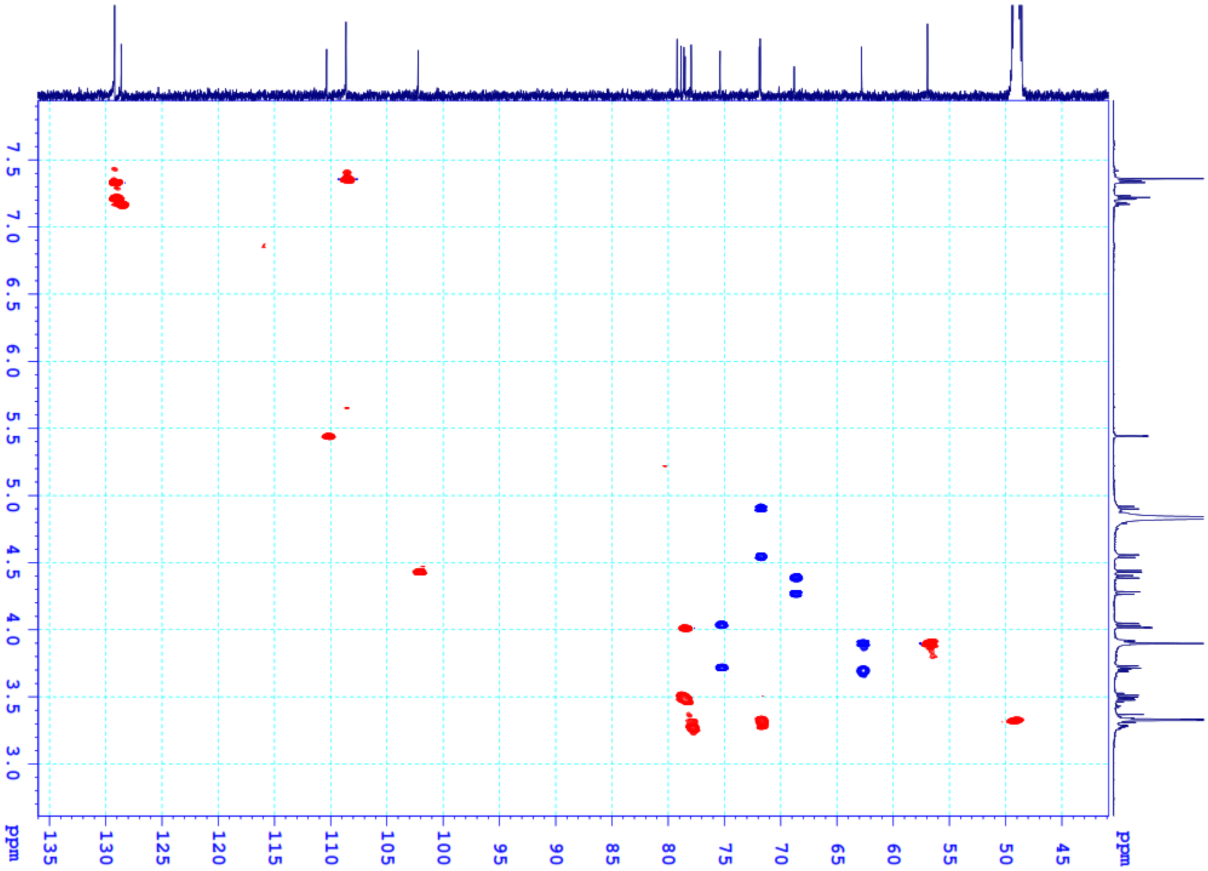

Figure S19. HSQC Spectrum of Compound **2**

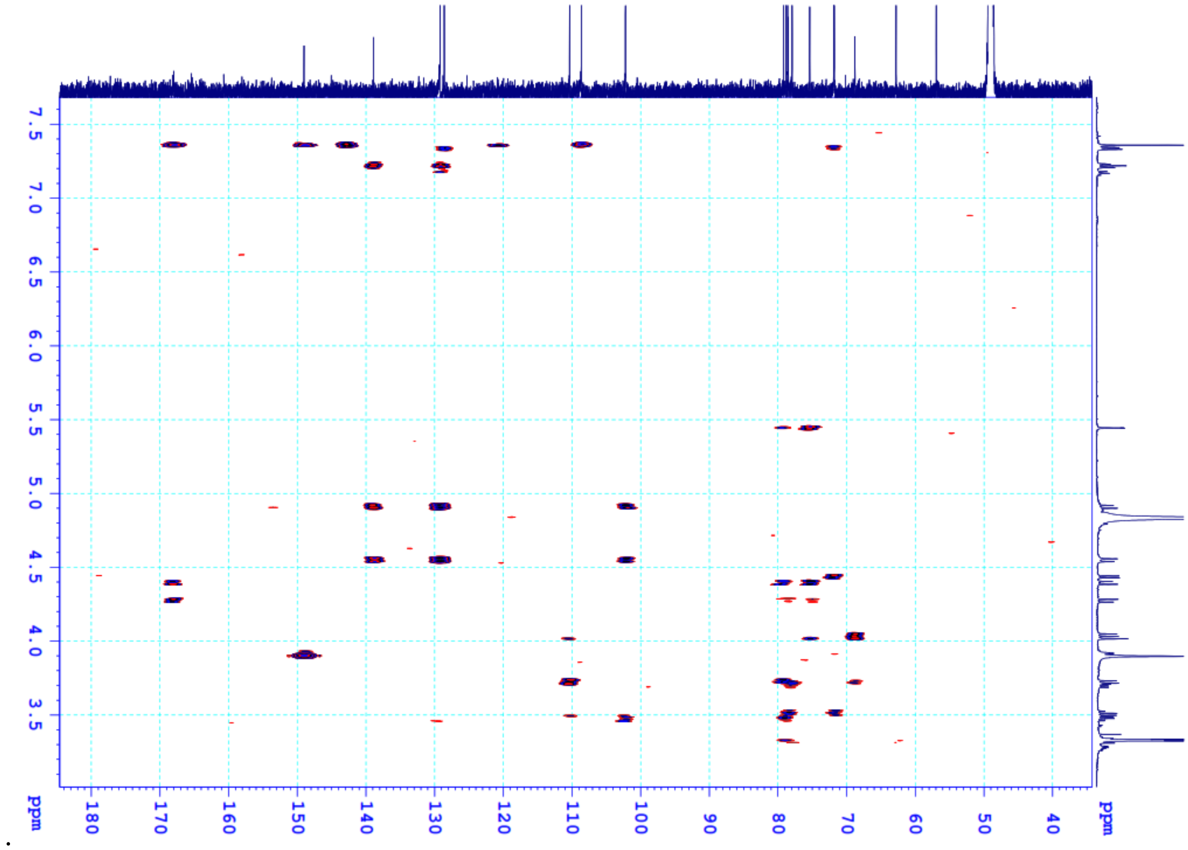

Figure S20. HMBC Spectrum of Compound **2**

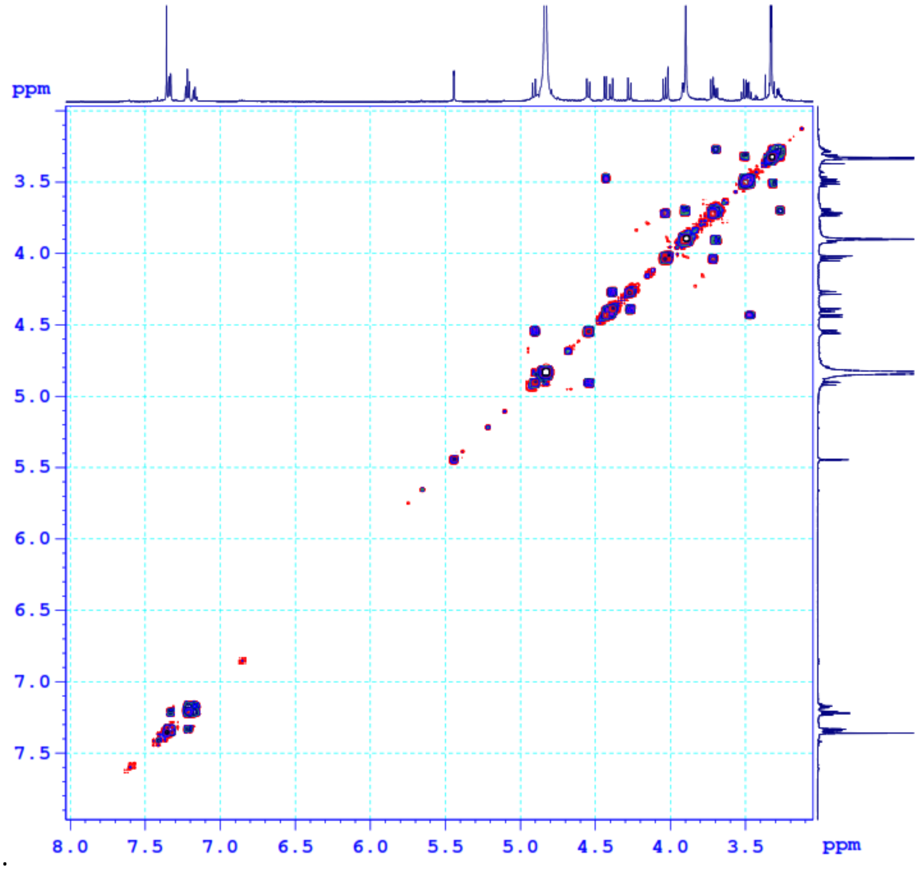

Figure S21. COSY Spectrum of Compound **2**
